# Leveraging Artificial Intelligence and Gene Expression Analysis to Identify Some Potential Bovine Coronavirus (BCoV) Receptors and Host Cell Enzymes Potentially Involved in the Viral Replication and Tissue Tropism

**DOI:** 10.3390/ijms26031328

**Published:** 2025-02-04

**Authors:** Mohd Yasir Khan, Abid Ullah Shah, Nithyadevi Duraisamy, Reda Nacif ElAlaoui, Mohammed Cherkaoui, Maged Gomaa Hemida

**Affiliations:** 1 Department of Computer Science, College of Digital Engineering and Artificial Intelligence, Long Island University, Brooklyn, NY 11201, USA; mohd.yasirkhan@liu.edu (M.Y.K.); nithyadevi.duraisamy@liu.edu (N.D.); reda.nacifelalaoui@liu.edu (R.N.E.); mohammed.cherkaoui@liu.edu (M.C.); 2 Department of Veterinary Biomedical Sciences, College of Veterinary Medicine, Long Island University, 720 Northern Boulevard, Brookville, NY 11548, USA; abidullah.shah@liu.edu

**Keywords:** BCoV, spike glycoprotein, hemagglutinin esterase, ACE-2, NPR1, Furin, TMPRRS2, docking, Neu5,9Ac2, homology modeling, virus/host interaction

## Abstract

Bovine coronavirus (BCoV) exhibits dual tissue tropism, infecting both the respiratory and enteric tracts of cattle. Viral entry into host cells requires a coordinated interaction between viral and host proteins. However, the specific cellular receptors and co-receptors facilitating BCoV entry remain poorly understood. Similarly, the roles of host proteases such as Furin, TMPRSS2, and Cathepsin-L (CTS-L), known to assist in the replication of other coronaviruses, have not been extensively explored for BCoV. This study aims to identify novel BCoV receptors and host proteases that modulate viral replication and tissue tropism. Bovine cell lines were infected with BCoV isolates from enteric and respiratory origins, and the host cell gene expression profiles post-infection were analyzed using next-generation sequencing (NGS). Differentially expressed genes encoding potential receptors and proteases were further assessed using in-silico prediction and molecular docking analysis. These analyses focused on known coronavirus receptors, including ACE2, NRP1, DPP4, APN, AXL, and CEACAM1, to identify their potential roles in BCoV infection. Validation of these findings was performed using the qRT-PCR assays targeting individual genes. We confirmed the gene expression profiles of these receptors and enzymes in some BCoV (+/−) lung tissues. Results revealed high binding affinities of 9-O-acetylated sialic acid and NRP1 to BCoV spike (S) and hemagglutinin-esterase (HE) proteins compared to ACE2, DPP4, and CEACAM1. Additionally, Furin and TMPRSS2 were predicted to interact with the BCoV-S polybasic cleavage site (RRSRR|A), suggesting their roles in S glycoprotein activation. This is the first study to explore the interactions of BCoV with multiple host receptors and proteases. Functional studies are recommended to confirm their roles in BCoV infection and replication.

## 1. Introduction

The virus replication occurs in many consecutive steps to generate many mature viral particles upon the completion of the viral replication in the permissive cells. The virus always hijacks the cellular machinery and directs them to synthesize their viral proteins instead of cellular ones [1,2]. The viral entry is a crucial step in any viral infection [3]. The viral entry requires the interaction between many proteins from the viral and host sides [1]. Viral-specific receptors are an important factor in viral entry into the host cells. The host cell is permissive for certain viruses if they express the specific viral receptors and provide suitable environments for virus replication, including any auxiliary receptors, transcription, and translation factors. Coronaviruses are enveloped viruses containing positive sense RNA genomes and belong to the order Nidovirales and are classified into four genera (α, β, λ, and δ) [4]. The beta (β) coronaviruses include five important human coronaviruses are the sever acute respiratory syndrome coronavirus (SARS-CoV), the sever acute respiratory syndrome coronavirus-2 (SARS-CoV-2), the Middel East respiratory syndrome coronavirus (MERS-CoV), human coronavirus-HKU1 (HCoV-HKU1) and the human coronavirus-OC43 (HCoV-OC-43). This genus includes some other important viruses affecting animals, particularly the bovine coronavirus (BCoV) and the equine coronavirus (ECoV) [4]. BCoV can cause significant health issues in cattle, leading to respiratory diseases and gastrointestinal problems, particularly in young calves. Economic impacts arise from decreased milk production, increased veterinary costs, and mortality in severe cases [5]. BCoV outbreaks can spread rapidly in herds, especially in crowded or poorly managed environments. Historical epidemics have highlighted the virus’s ability to cause widespread disease and stress in livestock populations. Researchers are investigating the genetic diversity of BCoV strains to better understand their evolution and potential for cross-species transmission and the mechanisms by which BCoV infects cells and causes disease, aiming to identify potential therapeutic targets [6]. The coronavirus’s genome size ranges from 27–31 kb and has a unique organization. The full-length genome is flanked by two untranslated regions at the 5′ and 3′ ends. Coronaviruses are characterized by producing a set of sub-genomic messenger RNA (mRNA) at their 3′ end [7]. The 5′ end of the genome of most coronaviruses contains a large gene called Gene-1, which consists of two overlapping open reading frames (ORFs) with a ribosomal frameshifting between those two ORFs. However, the 3′ end of the genome is mainly occupied by the common structural proteins interspersed with some small accessory proteins. There are four major structural proteins in most coronaviruses, including the spike glycoprotein (S), the envelope (E), the membrane (M), and the nucleocapsid protein (N). Some members of the genus β-coronavirus, including BCoV and HCoV-OC43, have an additional structural protein called hemagglutinin esterase (HE); thus, their genome is a little larger in size compared to other coronaviruses (31-kilobases) [8]. The S glycoprotein is a key player in all coronavirus replication. There are several proteins, including some cellular receptors, co-receptors, and cellular enzymes, that are involved in the BCoV/host interaction. The BCoV spike (BCoV/S) and BCoV/HE proteins are important in virus replication and pathogenesis [8]. BCoV-S has potential bidding to the 5-N-acetyl-9-O-acetylneuraminic acid, suggesting their possible roles as BCoV receptors [7]. On the other hand, the BCoV-HE acts as a receptor-destroying enzyme during BCoV replication [9]. However, there is a lack of comprehensive understanding of the interplay of the BCoV-S/BCoV-HE and the cellular receptors during BCoV replication. The availability of specific receptors is one of the main factors that make the target cells permissive to Coronavirus (CoVs) infection. Each group of CoVs recognizes certain types of receptors and may require the presence of additional auxiliary receptors to facilitate virus attachment and downstream replication. SARS-CoV-2 uses the angiotensinogen-converting enzyme-2 (ACE-2) as the main receptor, and the chaperone GRP78 acts as an auxiliary receptor [10,11]. It was also shown that MERS-CoV utilizes the dipeptidyl peptidase-4 (DPP4) as receptors in humans and dormitory camels [12,13]. The amino-peptidase N (APN), also called cluster of differentiation -13 (CD13), acts as a major receptor for the transmissible gastroenteritis virus that causes enteric infections in pigs [14]. The carcinoembryonic antigen cell adhesion molecule 1 (CEACAM-1) also acts as a major receptor to another coronavirus called murine hepatitis virus (MHV) [15]. Although the presence of the CoVs receptors and co/receptors is important for the success of viral replication, most coronaviruses require the presence of host cell enzymes that help in the cleavage of the CoV-S and CoV-HE proteins to initiate the process of viral infection. Usually, these host cell enzymes are enriched at the portal of entry of most coronaviruses, particularly the mucosal surfaces of the respiratory and enteric tracts of the affected hosts. It has been recently shown that TMPRSS2 (transmembrane protease serine 2) is a serine protease that plays an important role in SARS-CoV-2 replication, particularly during viral entry to the host cells. TMPRSS2 usually cleaves the viral spike glycoprotein, which activates the virus and facilitates its entrance to the host cells [16]. Furin is another host cell enzyme (subtilisin-like proprotein convertase). Furin usually cleaves the target proteins at a polybasic amino acid sequence R-X-(K/R)-R (where R is arginine, K is lysine, and X can be any amino acid). The furin cleavage to the SARS-CoV-2-S protein enhances the pathogenicity transmissibility and increases the virus infectivity in the target host [17,18]. Although the roles of the above-mentioned receptors and enzymes were intensively studied in SARS-CoV2, there is a lack of knowledge about the roles of these receptors and enzymes in BCoV infection and replication. Other than furin and TMPRSS2, some intracellular enzymes are also capable of cleavage and host pathogenicity to viruses. Cathepsin-L (CTS-L) is an intracellular enzyme and a member of the lysosomal cysteine protease family. CTSL cleavage promoted S to adopt receptor-binding domain (RBD) “up” activated conformations, facilitating receptor-binding and membrane fusion. The highly conserved CS-2 site seems more essential for the life cycle of SARS-CoVs, while CS-1 is likely to play an auxiliary role in host infection. The main goal of the current study is to use the in-silico prediction and gene expression investigation of different receptors and proteases of the bovine host to study the roles of these proteins and enzymes of BCoV in interaction with the host cell and its related pathogenicity. This study shed light on some unknown aspects of BCoV tissue tropism and pathogenesis. It will also pave the way for the development of some novel vaccines and antiviral therapies for BCoV infection in cattle.

The applications of artificial intelligence (AI) tools, particularly molecular docking and simulation, are a hot topic nowadays [19,20,21,22]. These tools have been widely used in many aspects of coronavirus research, including vaccine and diagnostic assay design, study of the roles of some viral/host proteins in the molecular pathogenesis of coronaviruses, design/repurposing of some antiviral drugs for the treatment of many coronaviral infections, particularly in humans, and identification of some novel receptors and enzymes for some coronaviruses [23,24]. AI has many advantages compared to the wet lab experiments in the above-mentioned research directions. First, the application of AI tools reduces the time spent screening compounds or designing vaccines. Second, AI design and more precision and accuracy of the designed models, including drugs and vaccines. Third, the application of AI tools may reduce the number of laboratory animals in research, which has great positive impacts on animal health. Fourth, AI tools eliminate some human errors in the design of some drugs or vaccines. However, we should not solely rely on AI for the drug/vaccine design for coronaviruses and other pathogens, and it is not realistic or scientifically sound to eliminate the wet lab validation and the animal experiments in this type of research. The best approach is to apply the combination of AI tools and the wet lab and functional validation using the proper number of animals to achieve the target goals in the precise vaccine or drug design for many viral diseases [25]. Our recent research showed the application of machine learning tools in the design of multi-epitope-based vaccines for BCoV [23].

## 2. Results

### 2.1. Differential Expression Pattern of Receptors in BCoV/Ent and BCoV/Resp Infected Cells In-Vitro

**Figure 1:**
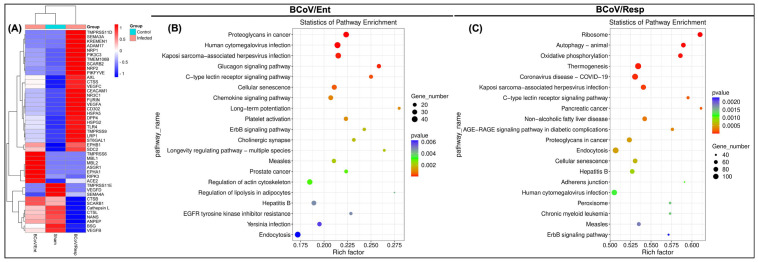
The bovine genes and pathways expression profiles during BCoV infection in cell culture (**A**) The Expression profiles of some host cell proteins during BCoV/Ent or BCoV/Resp Infection in some bovine cell lines: Heatmap showing the differentially expressed coronavirus-related receptors in the control (sham), BCoV/Ent and BCoV/Resp infected group of BEC cells based on the NGS data analysis. (**B**) The KEGG pathway analysis of next-generation sequencing (NGS) data indicates differentially expressed host cellular pathways during infection with the BCoV/Ent isolate and (**C**) during infection with the BCoV/Resp isolate. The Heatmap and KEGG pathway diagram was constructed using online bioinformatical software (https://www.bioinformatics.com.cn/en) on 9 November 2023.

Next-generation sequencing (NGS) was performed to examine the expression pattern of several known coronavirus receptors following infection with BCoV/Enteric (BCoV/Ent) and BCoV/Respiratory (BCoV/Resp) in BEC cells in-vitro. The results revealed that NRP1, AXL, CEACAM1, and Furin protease were downregulated in the BCoV/Ent infected group but upregulated in the BCoV/Resp infected group (Figure 1A). The VEGF receptor family, which interacts with the NRP1 complex, displayed differential expression in BCoV/Ent and BCoV/Resp infected groups. Specifically, VEGFA and VEGFC were upregulated in both infected groups, while VEGFB and VEGFD were downregulated compared to the sham. The EPHA1 receptor was upregulated in the BCoV/Ent infected group, while EBHB1 receptors were upregulated in both BCoV/Ent and BCoV/Resp infected groups compared to the sham. The TMEM106B and SDC2 were upregulated in both BCoV/Ent and BCoV/Resp infected groups compared to the sham. The expression of ACE2 was upregulated in the BCoV/Ent infected group, while no change was observed in the BCoV/Resp infected group (Figure 1A). The expression of CatapsinL was upregulated in the BCoV/Ent infected group and downregulated in the BCoV/Resp infected group (Figure 1A). These findings suggest that receptor expression varies depending on the BCoV isolates.

The KEGG pathway enrichment analysis of the NGS data identified significant virus-related pathways involved with the response to BCoV/Ent and BCoV/Resp infection (Figure 1B,C). In the BCoV/Ent infected group, KEGG pathway analysis revealed similarity in the pathways related to the Human Cytomegalovirus (HCMV), Kaposi sarcoma-associated herpesvirus, hepatitis B, and Yersinia infection pathways (Figure 1B). Meanwhile, the KEGG pathway analysis of the BCoV/Resp infected group indicated similarity with pathways involved in COVID-19, Kaposi sarcoma-associated herpesvirus, hepatitis B, HCMV, and Measles (Figure 1C). These results highlight distinct infection patterns between BCoV/Ent and BCoV/Resp isolates involved in distinct KEGG-related virus-specific pathways.

### 2.2. Mapping of the BCoV Spike Glycoprotein Structural Domains

**Figure 2:**
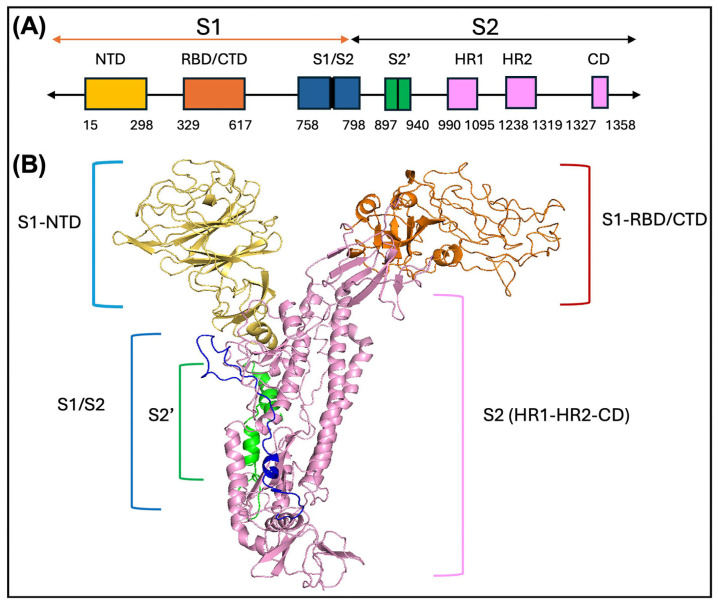
The schematic representations of the structure of the BCoV-spike glycoprotein: S1 and S2 Chains. (**A**) The listed domain boundaries are primarily defined as S-NTD, N-terminal Domain; RBD, Receptor Binding Domain; S1-CTD, C-Terminal domain; S2-HR1, Heptad Repeat 1; S2-HR2, Heptad Repeat 2 (**B**) Schematic drawing of the three-dimensional structure of BCoV Spike protein showing different domains on S1 and S2 chains.

In the BCoV-spike-protein sequence, the conserved domain region was identified through the PROSITE server and InterPro server. Based on the results of the PROSITE server and InterPro server, the BCoV spike glycoprotein has two chains: spike protein S1 and S2. The chain S1 contains two domains: an N-terminal galectin-like domain (NTD) and a receptor-binding domain (S1 RBD), also referred to as the RBD site present in between the S1 C-terminal domain (CTD) (Figure 2A,B). The NTD of S1 is of 15–298 amino acids. However, the S1-RBD site has 343–491 amino acid residues, which are present between the CTD of the S1 protein, and it is 329–617 amino acids long [26]. The amino acids from 629 to 1297 contain the S1/S2 cleavage region, the S2 fusion subunit of the spike (S) glycoprotein, and the HR1 and HR2 region from Betacoronaviruses (BCoV). The protease action related to the S1/S2 cleavage region is between 758–797 amino acid residues of the BCoV spike protein structure (Figure 2A,B).

### 2.3. The Homology Modeling of the BCoV-Spike Glycoprotein and Some Potential Coronavirus Host Cell Receptors

To perform the homology modeling, we searched for the experimentally determined template reference sequences closely related to the query sequence and shared at least 65% sequence identity. The BCoV spike protein sequence (Strain: Mebus) of 1363 amino acids (AA) in length. The sequences of the target molecules from bovine as a receptor are ACE2 (804 AA.), the host cell Furin (797 AA), the TMPRSS2 (490 AA), the NRP1 (924 AA), the DPP4 (765 AA), the APN (965 AA) and the CEACAM-1 (436 AA). Generally, the homology models, with lower DOPE scores and lower PDF total energy values, are considered more structurally stable and reliable. However, if the total energy values for multiple models are very similar, the DOPE score can be used as the deciding factor based on its statistical potential. Therefore, out of five, we selected the two best models with the PDF total energy values and lowest DOPE score for each protein homology model (Table S1).

Out of these five models, based on the Lowest DOPE score and PDF total energy, one of the best models that has been further considered for other parameters, such as the Ramachandran plot [27] and VERIFY 3D (Table 1) was used to assess the protein 3D model’s structural integrity.

The best BCoV spike protein model has a DOPE score of −137,057.54, and a PDF total energy value of 51,589.75 (model 1) was chosen in the case of the BCoV. The spike protein Ramachandran plot shows that the maximum of the total amino acid residues is in the most favored regions and then is in the additional most allowed areas. A small proportion of residues are in the generously allowed regions. Based on Ramachandran plot results, all the predicted models, including BCoV/S, ACE2, NRP1, TMPRSS2, Furin, CTS-L CEACAM-1, DPP4, AXL, and APN, indicate a highly accurate homology model of each structure (Figure S1).

Therefore, the Ramachandran plot and Verify-3D score suggest that the modeled proteins are stable, and the score is closer to the expected high score (Table 1), showing that the predicted homology models of all protein structures are of good quality.

### 2.4. Docking of the Bovine Neu5,9Ac2 with the BCoV Spike and Hemagglutinin Esterase (HE) Protein

**Figure 3:**
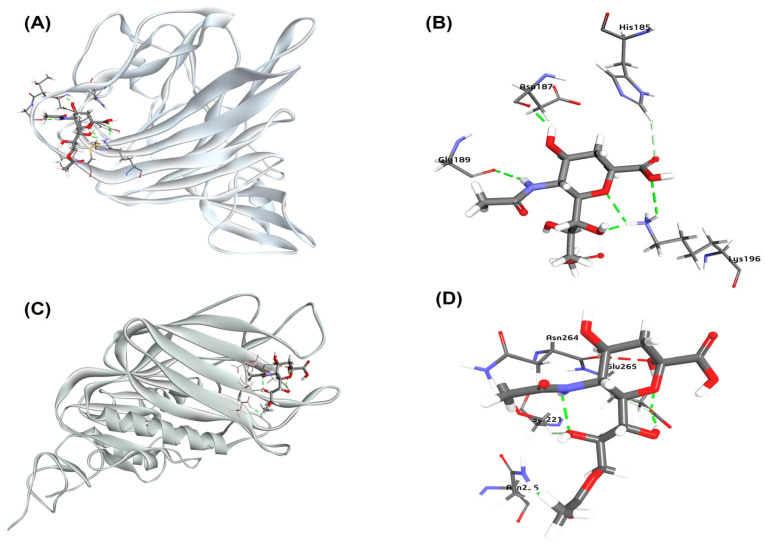
Sialic acid (Neu5,9Ac2) as the receptor for BCoV Spike at the NTD site and HE protein. (**A**) Neu5,9Ac2 binding to the N-terminal domain (NTD) of the BCoV Spike protein is illustrated. (**B**) Detailed views of the interactions between Neu5,9Ac2 and the specific amino acid residues at the NTD site of the BCoV Spike protein: His185, Asp187, Gly189, and Lys196. (**C**) the binding of Neu5,9Ac2 as a ligand for the BCoV/HE protein receptor. (**D**) showing ligand Neu5,9Ac2 binding to the interacted amino acid residues- (Asn-264, Glu-265, Ser-221, and Asn-236) of the BCoV/HE protein. The dotted green lines show hydrogen bonding, the red dotted lines are pi-interactions, and the white dotted lines are conventional hydrogen bonds between the ligand and amino acid residues of BCoV/S-NTD and HE protein.

It was previously reported that the Neu5,9Ac2 is a receptor for BCoV/S-NTD [28]. To investigate the molecular interaction of the BCoV/S with the bovine cell surface receptor molecule, we performed molecular docking of the BCoV/S-NTD (PDB ID: 4H14) with molecule Neu5,9Ac2. The docking result gives ten poses of ligand-protein interaction complexes. Further, ligand-protein interaction binding energy (BE) calculation was done to calculate the binding energy of complexes. Binding affinity determines the interaction and stability of any given protein-ligand complex or the binding affinity of a ligand to a protein. The binding energy calculation result of the ten best ligand-protein complexes indicated a strong binding affinity between the Neu5,9Ac2 molecule and the BCoV/S-NTD. The interaction between Neu5,9Ac2 and BCoV/S NTD with negative binding energy (−79.55 kcal/mol) is shown as the best ligand-protein interaction pose in Figure 3. This interaction involves some key amino acid residues, including Asp-187, Gly-189, His-185, and Lys-196 (Figure 3A,B). The docking results revealed that Neu5,9Ac2 interacts with a specific pocket above the β-sandwich core in the BCoV/S-NTD.

Furthermore, the X-ray crystal structure of the BCoV/HE complex with the sialic acid retrieved from the RCSB PDB database (PDB ID:3CL5; 1.80 Å resolution) and was chosen as the receptor for ligand Neu5,9Ac. The docking of Neu5,9Ac2 with HE indicated a strong binding affinity between the sugar molecule and the BCoV/HE, with the highest binding energy (−122.73 Kcal/mol). The docking of Neu5,9Ac2 results suggested its interaction with a specific pocket where sialic acid interacts with the HE. This interaction involves critical amino acid residues, including Asn-264, Glu-265, Ser-221, and Asn-236 (Figure 3C,D).

### 2.5. The Interaction Between Heparan Sulphate (HSPG2) and the BCoV/S Glycoprotein

**Figure 4:**
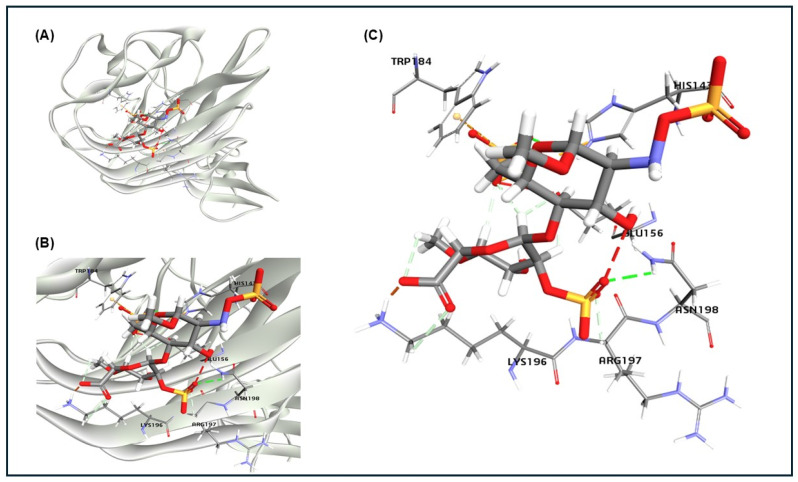
A proposed model for the bovine heparan sulphate (HS) of HSPG2 interaction with the BCoV/S protein. (**A**) shows the binding of HS with the BCoV/S protein receptor. (**B**,**C**) showing ligand binding to the interacted amino acid residues- (TRP184, HIS143, GLU156, ASN198, ARG197, and LYS196 of the BCoV/S protein. The dotted green lines show hydrogen bonding, the red dotted lines are pi-interactions, and the white dotted lines are conventional hydrogen bonds between ligand HS and amino acid residues of BCoV/S-NTD.

The docking of glycan compound heparan sulphate (HS) from proteoglycan protein HSPG2 interacted with BCoV/S-NTD (PDB ID: 4h14). The docking result gives ten poses of ligand-protein interaction complexes. The interaction indicated a strong binding affinity between the HS molecule and the BCoV/S, with the highest binding affinity (−43.27 Kcal/mol). The HS interaction involves critical amino acid residues from BCoV/S, including TRP184, HIS143, GLU156, ASN198, ARG197 and LYS192 (Figure 4A–C). The docking (molecular interaction) result of the BCoV/S with the HS glycan of the HSPG2 receptor from the bovine cell surface showed high binding affinity towards both the spike protein of BCoV.

### 2.6. BCoV Spike Glycoprotein and the Bovine ACE2 Protein

**Figure 5:**
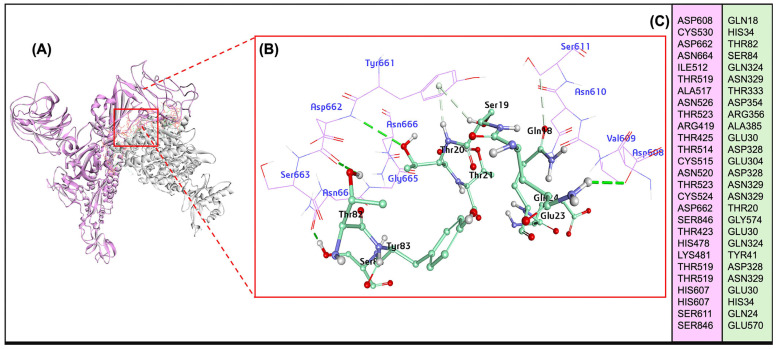
A proposed Model for the BCoV/S glycoprotein interaction with the bovine ACE2 protein. (**A**) The BCoV Spike protein interacts with ACE2, showing possible binding interactions at the receptor-binding domain (RBD) of the C-terminal domain (CTD) of the S1 protein chain. (**B**,**C**) Detailed views of the protein-protein interaction interface, highlighting the amino acid residues involved in the interaction between ACE2 and the N-terminal domain (NTD) of the BCoV Spike protein. ACE2 interaction residues: Ser19, Thr20, Thr21, Glu23, Gln24, Thr27, Glu30, Lys81, Thr82 and other residues. BCoV Spike NTD interaction residues: Tyr661, Asp662, Ser663, Gly665, Asn666, Ser611, Thr425, Arg419, Ala527 and other residues. The dotted green lines show hydrogen bonding, the red dotted lines are pi-interactions, and the white dotted lines are conventional hydrogen bonds between ACE2 and amino acid residues of BCoV/S protein.

The energy minimization for the bovine ACE2 protein, the BCoV spike protein, and protein-protein dockings were performed on the ZDOCK server. The ACE2 interacted putatively with the BCoV-spike N terminal domain of the S1 protein based on the homology-modeled BCoV spike protein. The ACE2/BCoV spike protein-protein interaction interface residues are given in (Figure 5A–C and Table S2). The total interaction energy between contact residues of bovine ACE2 and the BCoV/S were computed and reported in units of kcal/mol. The best docking pose shows a ZDOCK interaction score of 34.52 and an E_RDock score of −6.26 kcal/mol. The molecular interaction of the ACE2 shows interaction with BCoV/s at the receptor binding domain (RBD) of the C-terminal domain (CTD) of the S1 protein chain. ACE2 interaction residues are SER19, THR20, THR21, GLU23, GLN24, THR27, GLU30, LYS81, THR 82 and other residues (Table S2). Spike S protein CTD-RBD site residues involved in interaction are TYR661, ASP662, SER663, GLY665, ASN666, SER611, THR425, ARG419, ALA527 and other residues (Table S2). The net interaction between ACE2 and the spike protein is attractive, as indicated by the negative binding Affinity score of interacted residues (RDOCK interaction energy) (Table 2).

Specifically, the receptor-protein interaction analysis showed differences in the key residues at the interface between ACE2 and S1 protein CTD (Figure 3). This might be due to the local sequence difference of BCoV spike protein among different species, suggesting that bovine ACE2 is a putative receptor for BCoV-spike due to low binding affinity (−6.26 kcal/mol) towards RBD or domain B of BCoV spike protein.

### 2.7. The Proposed Model for the Interactions Between the Bovine NRP1 Protein and the BCoV/S Glycoproteins

To test the potential roles of the bovine NRP1-as novel receptors for BCoV, the ZDock docking method of protein-protein interaction was performed to identify the interaction sites of the NRP1 with BCoV spike protein.

**Figure 6:**
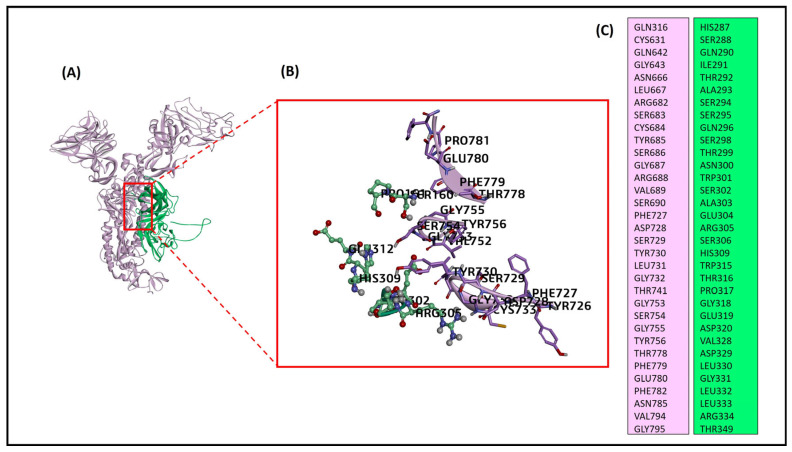
The proposed model for the interaction between the bovine NRP1 and the BCoV/S glycoprotein. (**A**) NRP1 interaction at the furin-specific recognition site (RRSRR region) at the BCoV/S1/S2 junction. (**B**,**C**) Conformation of the NRP1 residues SER302, ALA303, GLU304, ARG305, SER306, HIS309, TRP315, THR316 and PRO317 with binding affinity to BCoV/S1 protein amino acid residues including THR741, GLY753, SER754, GLY755, TYR756, THR778, PHE779, GLU780, PHE782 and ASN785 near RRSRR|A (S1/S2) cleavage sites. The dotted green lines show hydrogen bonding, the red dotted lines are pi-interactions, and the white dotted lines are conventional hydrogen bonds between NRP1 and amino acid residues of BCoV/S.

The protein-protein interaction of the bovine host cell NRP1 and the BCoV/S glycoprotein suggested the potential binding of NRP1 at the S1/S2 site identified by the ZDOCK Score 23.72 and E_RDock score −15.14 kcal/mol. Among the ten refined poses from E_RDock, the best pose of the NRP1 binding to the S1/S2 region exhibited near the protease cleaving site of polybasic amino acid residues (Arg764, Arg765, Ser766, Arg767, Arg768, and Ala 769 (RRSRRA) at BCoVS1/S2 cleavage sites (Figure 6A–C). The protein NRP1 interacted with spike protein at the amino acid residues THR741, GLY753, SER754, GLY755, TYR756, THR778, PHE779, GLU780, PHE782 and ASN785. These residues are present upstream and downstream to the spike protein S1/S2 cleavage RRSRRA site. The residues of NRP1 involved in interaction are SER302, ALA303, GLU304, ARG305, SER306, HIS309, TRP315, THR316 and PRO317. However, the complete interaction site of both BCoV/S-NRP1 is shown in Figure 6C. The total number of hydrogen bonds and other non-covalent bonds between NRP1 and BCoV/S suggested greater and more stable binding between receptor and spike protein (Table 2).

### 2.8. BCoV/S Glycoprotein Interaction with the CEACAM1

**Figure 7:**
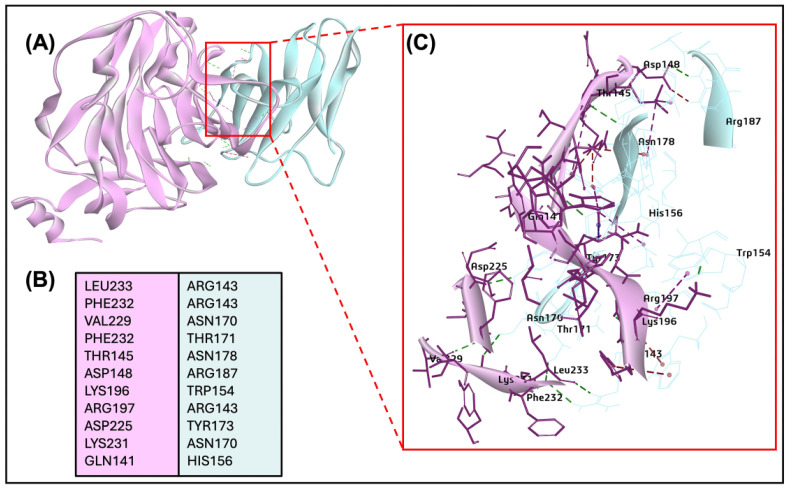
A putative model for the interaction between the bovine CEACAM-1 protein and the BCoV-S glycoprotein and mapping the interactive amino acid residues. (**A**) The bovine CEACAM-1 protein interacts with the BCoV/S glycoprotein NTD of the BCoV spike. (**B**,**C**) CEACAM-1 binds through LEU233, PHE232, VAL229, Phe232, THR145, ASP148, and other amino acids with N-terminal domain (NTD) of Spike glycoprotein with residues ARG143, ASN170, THR171, ASN178, LYS196, LYS196 with hydrogen bonds. The dotted green lines show hydrogen bonding, the red dotted lines are pi-interactions, and the white dotted lines are conventional hydrogen bonds between CEACAM-1 and amino acid residues of BCoV/S.

To test the potential use of CEACAM1 as a receptor for the BCoV, the ZDock docking method of protein-protein interaction was performed to map the interaction sites of the CEACAM1 with BCoV/S glycoprotein. Our results show that the bovine CEACAM-1 protein binds with the BCOV-S/N-terminal domain (NTD). The interacting interface of amino acid residues for both proteins is shown in (Figure 7A–C and Table S2). The CEACAM1 protein shows a high binding specificity with the BCoV/S1-NTD site of Spike glycoprotein with residues ARG143, ASN170, THR171, ASN178, LYS196, LYS196, and other amino acid residues given in the (Table S2). The residues from BCoV spike S1-NTD that interacted with CEACAM1 are LEU233, PHE232, VAL229, Phe232, THR145, ASP148, and other amino acid residues shown in Table S2. The best dock pose shows a ZDOCK interaction score of 16.8, and E_R Dock interaction affinity is −6.29 kcal/mol due to multiple non-covalent interactions, including nine conventional hydrogens bonds, 12 Pi bond, and one salt bridge interaction between the two proteins (Figure 7A–C and Table 2). Therefore, the results suggested the binding of BCoV/S-NTD towards CEACAM1 with lower binding affinity than NRP1. However, the complete interaction site of both BCoV/S-CEACAM1 is shown in Figure 7C.

### 2.9. The BCoV/S Glycoprotein Interaction with the Bovine Aminopeptidase N (APN) Protein

**Figure 8:**
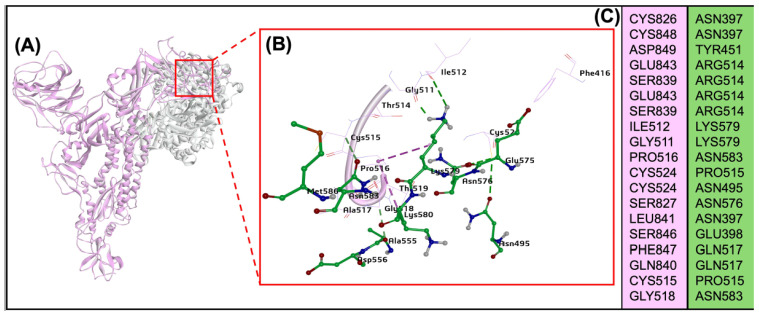
A putative model representing the interaction of BCoV/S glycoprotein and the bovine aminopeptidase N (APN) protein. (**A**) The APN interactions with BCoV/S1 RBD and CTD (**B**,**C**) BCoV/S1 chain CTD residues binding to the bovine APN through the residues TYR412, PHE416, HIS478, CYS524, ILE512, GLY511, THR514, CYS515, PRO516, THR518, ALA517, and GLY518 to amino acid residues ASN495, ALA555, ASP565, GLY575, ASP576, ARG614 and other amino acid residues of APN by hydrogen bonds. The dotted green lines show hydrogen bonding, red doted lines are pi-interactions and white dotted lines are conventional hydrogen bonds between APN and amino acid residues of BCoV/S.

To explore the potential roles of the bovine APN as a receptor for the BCoV, the ZDock with RDOCK docking method of the protein-protein interaction was performed to map the BCoV spike protein interaction sites with the bovine APN protein. The interaction between the two proteins suggested that the APN showed binding with the RBD of the BCoV/S glycoprotein. The interacting interface amino acid residues for both proteins are shown in (Figure 8A–C). The APN protein shows a binding specificity with RBD present in the BCoV/S1-CTD site of BCoV/S glycoprotein through the residues ASN397, TYR451, ARG514, LYS579, ASN583, CYS524, and other amino acid residues given in (Table S2). The residues of BCoV/S1-CTD interacted with the APN are CYS826, ASP849, GLU843, SER839, GLU843, SER839, ILE512, GLY511, PRO516, ASN495 and other amino acid residues given in (Table S2). The best docking pose with low-energy binding conformation shows a ZDOCK interaction score 17.6 and an E_RDock score of −2.96 kcal/mol. The interaction between proteins is due to multiple non-covalent bonds, including 19 conventional hydrogen bonds, two Pi bonds, and one salt bridge interaction between the two proteins (Figure 8A,B, and Table 2). However, the complete interaction site of both BCoV/S-APN is given in Figure 8C.

### 2.10. BCoV/S Glycoprotein Interaction with the Bovine Dipeptidyl Peptidase 4 (DPP4) Protein

**Figure 9:**
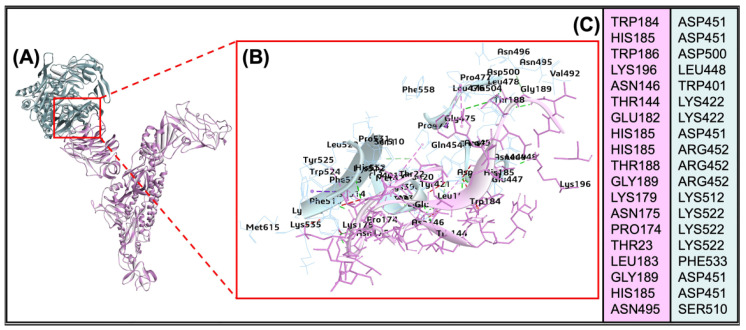
A proposed model for the interaction between the bovine DPP4 protein and the BCoV-S glycoprotein. (**A**) The DPP4 protein interacts with the BCoV/S1 protein NTD. (**B**,**C**) The BCoV/S1 chain N-terminal domain (NTD) residues bind to the DPP4 through TRP184, HIS185, TRP186, ASP187, THR188, GLY189, LYS196 which is core site for Neu5, 9Ac2 binding to BCoV-NTD. The binding amino acid residues from DPP4 to the NTD site are TYR43, VAL441 ARG552 GLY475, LEU476, LYS-512, PHE515, LYS520, HIS532, PHE533, LYS535, MET615 and other amino acid residues by hydrogen bonds. The dotted green lines show hydrogen bonding, red dotted lines are pi-interactions, and white dotted lines are conventional hydrogen bonds between DPP4 and amino acid residues of BCoV/S.

The bovine DPP4 protein interacts with the CTD of the BCoV/S protein. The amino acid residues at the interacting interface for both proteins are detailed in (Table S2) and illustrated in (Figure 9A–C). The DPP4 protein exhibited binding specificity with the receptor-binding domain (RBD) in the BCoV/S-CTD region, involving residues TRP184, HIS185, TRP186, LYS196, TRP401, LYS422, along with other interacting amino acids listed in (Table S2). The residues of the BCoV/S-CTD that interacted with the bovine DPP4 protein are (ASP451, ASP500, LEU448, ASN146, THR144, GLU182, HIS185, THR188, GLY189, and other interacted amino acid residues given in the (Table S2). The best docking pose shows a ZDOCK interaction score 22.44 and an E_R Dock score of −6.23 kcal/mol. These parameters might be due to multiple non-covalent interactions, including 20 conventional hydrogen bonds, nine Pi bonds, and one salt bridge interaction between the two proteins (Table 2). DPP4 shows interaction to CTD of BCoV/S with lower binding affinity than NRP1 towards BCoV/S.

### 2.11. BCoV/S Glycoprotein Interaction with the Bovine AXL Protein

**Figure 10:**
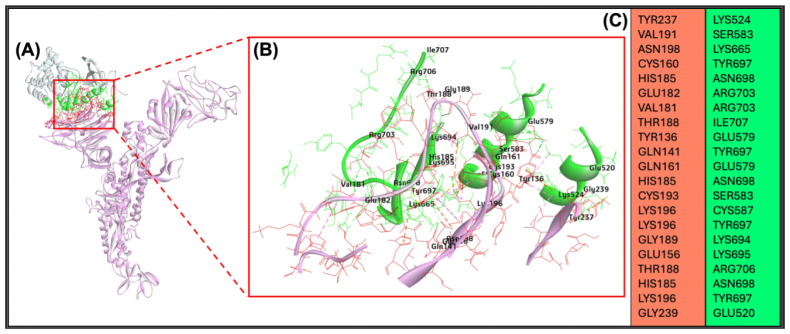
A proposed model for the interaction between the bovine AXL protein and the BCoV/S glycoprotein. (**A**) The AXL protein interacts with the BCoV/S1 protein NTD. (**B**,**C**) The BCoV/S1 chain N-terminal domain (NTD) residues bind to the AXL through TYR237, VAL191, ASN198, CYS160, HIS185, GLU182, VAL181, THR188, TYR136, GLN141, GLN161, CYS193, LYS196, GLY189, GLU156 and GLY239. The binding amino acid residues from AXL to the NTD site are LYS524, SER583, LYS665, TYR697, ASN698, ARG703, ILE707, GLU579, CYS587, LYS694, LYS695, ARG706 residues through hydrogen bonds. The dotted green lines show hydrogen bonding, the red dotted lines are pi-interactions, and the white dotted lines are conventional hydrogen bonds between AXL and amino acid residues of BCoV/S. highlighted with green in the figure legend.

The in silico docking procedure reveals the potential profound interactions between AXL and BCoV/S. The bovine AXL protein binds to the NTD of the BCoV/S protein. The interacting interface of amino acid residues for both proteins is shown in (Table S2 and Figure 10A–C). The AXL protein showed binding specificity with NTD in the BCoV/S with the residues LYS524, SER583, LYS665, TYR697, ASN698, ARG703, ILE707, GLU579, CYS587, LYS694, LYS695, ARG706. The residues of the BCoV/S1-NTD interacted with the bovine AXL protein are TYR237, VAL191, ASN198, CYS160, HIS185, GLU182, VAL181, THR188, TYR136, GLN141, GLN161, CYS193, LYS196, GLY189, GLU156 and GLY239. The best docking pose shows a ZDOCK interaction score of 17.56 and an E_R Dock score of -13.68 kcal/mol. The AXL shows high binding/interaction energy with BCoV/S due to multiple non-covalent interactions, including 21 hydrogen bonds and nine Pi bonds between the two proteins (Table 2).

### 2.12. A Protein-Protein Interaction of Bovine Proteases-Furin, TMPRSS2, and Cathepsin-L (CTS-L) with the BCoV/S

The online ProP 1.0 Server software prediction showed that one pro-peptide cleavage site, Arg764, Arg765, Ser766, Arg767, Arg768, and Ala 769 (RRSRRA) amino acids, is present across the full-length sequence of the BCoV/S (Uni-prot ID: P15777) (Figure S2).

**Figure 11:**
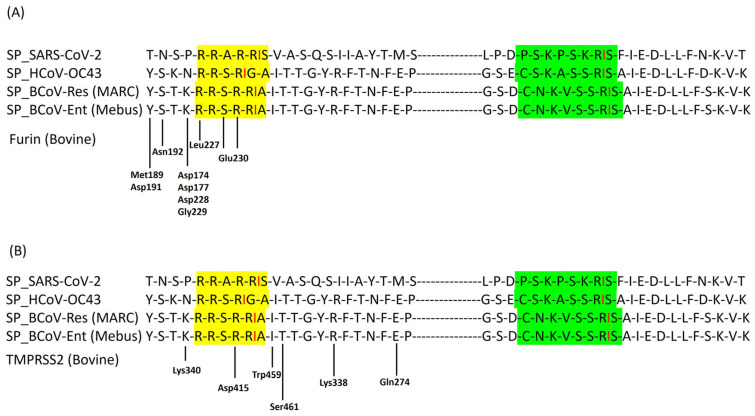
The multiple sequence alignment of the BCoV/S enteric (Mebus) strain cleavage site with some other human and animal coronaviruses: The yellow highlighted region contains polybasic residues and its specific cleavage site for furin S1/S2 junction (RRSRR) and S2′ site (CNKVSSRS) specific for TMPRSS2 protease. (**A**) the furin protease binding residues near its cleavage (RRSRRA) at the S1/S2 site and of the S protein. (**B**) showing the TMPRSS2 protease binding residues near its cleavage (RRSRR) at the S1/S2 site of S protein. The region highlighted with yellow is RRSRRA (S1/S2) present in BCoV/S, which is specific for cleavage sites for furin. The region highlighted with green is CNKVSSRS (S2′ ) present in BCoV/S, which is specific for the cleavage site for TMPRSS2. The red | is the site of the bond between amino acids specific for cleavage for proteases.

The multiple sequence alignments of the BCoV/S (Mebus) sequence with other Beta- coronaviruses spike protein sequences suggested protease-specific residues are Arg764, Arg765, Ser766, Arg767, Arg768, and Ala 769 (RRSRRA) (Figure 11A and Figure S3). The S2′ is the cleavage site for TMPRSS2 of BCoV/S, which is presented between amino acid sequences from 907–914 (CNKVSSRS) (Figure 11B). This finding suggests that host bovine proteases such as Furin and TMPRSS2 enzymes could cleave at these polybasic amino acid residue sites.

**Figure 12:**
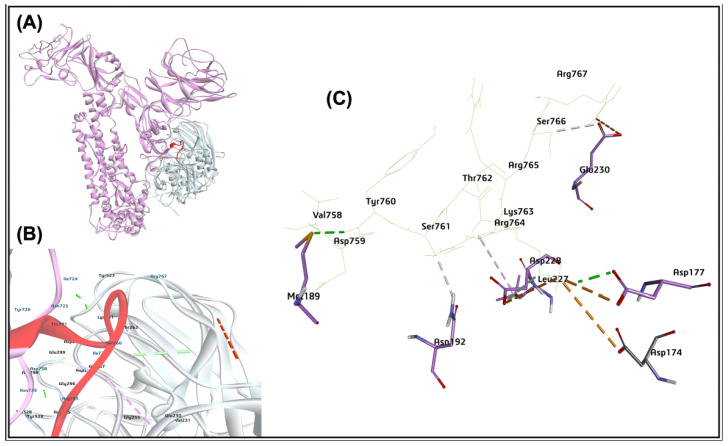
The proposed model of furin protease interactions with BCoV/S cleavage sites. (**A**) Furin protease interaction at the furin-specific recognition and cleavage site is highlighted with red (RRSRR|A region) at the BCoV/S1/S2 junction. (**B**,**C**) Conformation of the furin residues Glu230, Leu227, Asp228, Asp174, Asp177, Asn192, Met189 with binding affinity to BCoV/S1 protein amino acid residues including Val758, Asp759, Tyr760 Ser761, Thr762 Lys763, Arg764, Arg765, Ser766, Arg767 at RRSRR|A (S1/S2) cleavage sites. The protein-protein interaction interface of BCoV/S-Furin residues is shown in (Table S3). The dotted green lines show hydrogen bonding, the red dotted lines are pi-interactions, and the yellow dotted lines are conventional hydrogen bonds between furin and amino acid residues of BCoV/S. highlighted with green in the figure legend.

The protein-protein interaction of the bovine host cell furin and the BCoV/S glycoprotein suggested the potential binding of furin at the cleavage site identified by the ZDOCK Score 16.72 and E_RDock score −13.14 kcal/mol. Among the ten refined poses from E_RDock, the best pose of the furin binding to the S1/S2 region mapped near the protease cleaving site of polybasic amino acid residues (Arg764, Arg765, Ser766, Arg767, Arg768, and Ala 769 (RRSRRA) at BCoVS1/S2 cleavage sites (Figure 12A–C). The protease furin interacted with spike protein at the amino acid residues (Glu230, Leu227, Asp228, Asp174, Asp177, Asn192, Met189) to the spike protein S1/S2 cleavage RRSRRA site amino acid residues (Val758, Asp759, Tyr760 Ser761, Thr762 Lys763, Arg764, Arg765, Ser766, and Arg767) (Figure 12A–C).

The protein-protein interface interacts with residues Lys736 from BCoV/S, forming an electrostatic interaction and a salt bridge interaction with ASP174, ASP177, and Asp228 residues of Furin. The Arg767 and Ser766 of the BCoV/S RRSRR cleavage site show an electrostatic interaction and hydrogen bonding to the Glu230 of the Furin (Table 2). The results of furin interaction with a high binding affinity score suggest its catalytic role at the S1/S2 junction, which plays a role in virus replication and pathogenicity.

**Figure 13:**
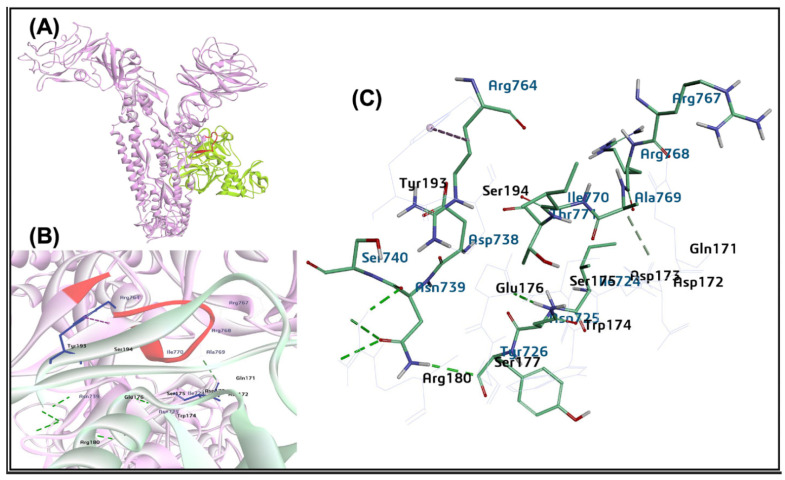
A proposed model representing the TMPRSS2 serin protease binding sites across the BCoV-S glycoprotein. (**A**). Mapping the binding sites upstream and downstream of the BCoV/S protein polybasic cleavage site RRSRR|A highlighted with red. (**B**,**C**) Mapping the TMPRSS2 residues (Val278, His294, Thr339, Asp415, Asn416, Trp459, Gly460, Ser461, Gly46)2 binding affinity to the BCoV/S1/S2 site (Asp738, Ser740, Thr741, Ser742, Ser743, Ser761, Arg764, Thr762, Lys763, Arg767, Thr771 and Ile779). The dotted green lines show hydrogen bonding, the red dotted lines are pi-interactions, and the white dotted lines are conventional hydrogen bonds between the TMPRSS2 and amino acid residues of BCoV/S.

The protein-protein interaction takes place between the bovine TMPRSS2 amino acid residues Val278, His294, Thr339, Asp415, Asn416, Trp459, Gly460, Ser461, Gly462 near the RRSRR site of BCoV/S protein residues (Asp738, Ser740, Thr741, Ser742, Ser743, Ser761, Arg764, Thr762, Lys763, Arg767, Thr771 and Ile779). The protein-protein docking between the BCoV/S/and TMPRSS2 in the ZDOCKER shows E_RDock energy by −0.62 kcal/mol due to multiple non-covalent interactions (conventional hydrogen and Pi bond) between the two proteins (Table 2 and Table S3). Among the top 10 refined poses, the best pose of the TMPRSS2 binding at the active sites (substrate binding site and catalytic site) of the BCoV/S2 protein is shown in (Figure 13A–C). In terms of the best pose binding affinity of furin, TMPRSS2 shows less binding affinity towards BCoV/S (Table 2).

**Figure 14:**
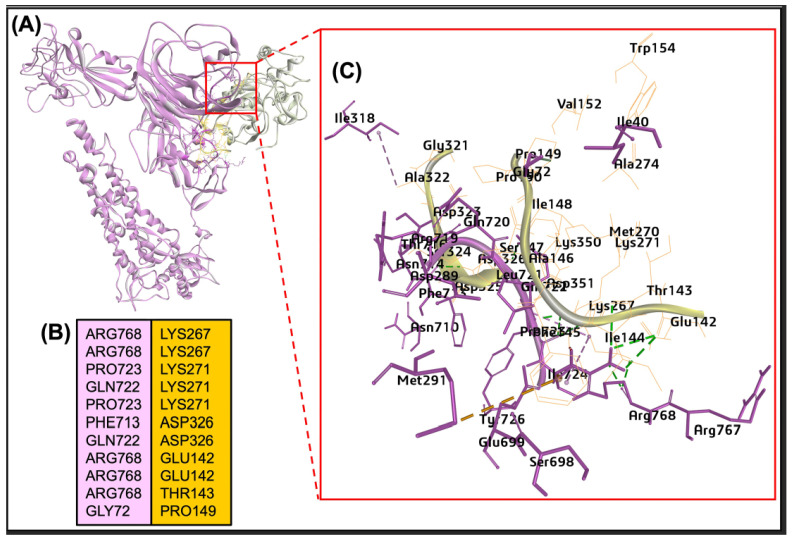
The image representing the CTS-L protease binding at NTD and CTD of the S1 chain from BCoV/S. (**A**) Mapping the interaction site of the BCoV/S and CTS-L protein (**B**,**C**) Mapping the interaction interface of CTS-L residues (Ile36, Met291, Thr38, Ala33, Ser35, Ile36, Gl72, Arg768, Pro723, Gln722, Phe713) binding affinity to the BCoV/S1 chain residues (Trp154, Phe145, Lys267, Thr275, Asp326, Gly190, Phe186, Tyr159, Asp326, Glu142, Thr143 and Pro149). The dotted green lines show hydrogen bonding, the red dotted lines are pi-interactions, and the white dotted lines are conventional hydrogen bonds between CTS-L and amino acid residues of BCoV/S.

The spike protein S1 chain of SARS-CoV-2 contains two highly conserved CTS-L cleavage sites (CS-1 and CS-2) among all known SARS-CoV-2 variants The multiple sequence alignment of BCoV/S from the Mebus strain suggested no conserved cathepsin-L cleavage site (CS-1 and CS-2) or region present in S chain of spike protein as SARS-CoV-2 (Figure S4). In the case of CTS-L protease, the interaction of the bovine host cell CTS-L and the BCoV/S glycoprotein suggested the potential binding site identified by the ZDOCK Score of 19.84 and E_RDock score of −16.45 kcal/mol. Among the ten refined poses from E_RDock, the best pose is the CTS-L binding to the residues of NTD and the downstream of the S1/S2 region of polybasic amino acid residues. The BCoV/S amino acid residues in this interaction through hydrogen bonding are Thr38, Ala33, Ser35, Ile36, Gl72, Arg768, Pro723, Gln722, Phe713 (Figure 14A–C). The Pi-interaction residues from BCoV/s are Ile36 and Met291. The protease CTS-L interacted with spike protein through amino acid residues Lys267, Thr275, Asp326, Gly190, Phe186, Tyr159, Asp326, Glu142, Thr143 and Pro149 (Figure 14A–C). The Pi-interaction residues from CTS-L are Trp154 and Phe145. The Arg786 residue from the polybasic RRSRR site at the S1/S2 junction of BCoV/S forms hydrogen bonding with the Lys276 residue of CTS-L. The results suggested the greater binding affinity of CTS-L towards the BCoV/S protein.

### 2.13. Differential Expression Pattern of Host Cell Surface Receptors upon BCoV/Ent and BCoV/Resp Infection In-Vitro

**Figure 15:**
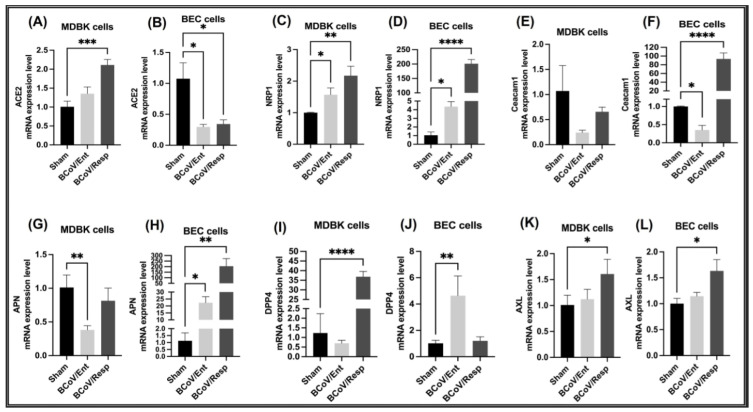
Assessment of the expression of the Bovine (ACE2, NRP1, CEACAM1, APN, DPP4, and AXL) mRNA expression profiles in the BCoV infected the MDBK and the BEC cells using qRT-PCR. (**A**,**C**,**E**,**G**,**I**,**K**) mRNA expression in MDBK cells infected with BCoV/Ent or BCoV/Resp isolates, compared to the sham, analyzed by qRT-PCR. (**B**,**D**,**F**,**H**,**J**,**L**) Compared to the sham, the mRNA expression in BEC cells infected with BCoV/Ent or BCoV/Resp isolates was analyzed by qRT-PCR. Both cell lines were infected with 1 MOI of either BCoV/Ent or BCoV/Resp isolates for 72 hpi and used for qRT-PCR analysis, (* *p* < 0.05, ** *p* < 0.01, *** *p* < 0.001, **** *p* < 0.0001).

The mRNA expression of bovine cell surface receptors was analyzed via qRT-PCR in host MDBK and BEC cells following infection with BCoV/Ent and BCoV/Resp isolates. In MDBK cells, ACE2 mRNA was upregulated in both infected groups, with a more pronounced increase observed in the BCoV/Resp group (Figure 15A). In BEC cells, the ACE2 mRNA expression was significantly downregulated in both the BCoV/Ent and BCoV/Resp infected groups compared to the sham (Figure 15B). This indicates that ACE2 may play a distinct role in BCoV/Ent and BCoV/Resp infections, depending on the cell type.

In MDBK cells, NRP1 mRNA was significantly upregulated in both the BCoV/Ent and BCoV/Resp infected groups compared to the sham (Figure 15C). Similarly, NRP1 mRNA expression was significantly increased in BEC cells in both infected groups compared to the sham (Figure 15D). Notably, NRP1 expression was higher in both MDBK and BEC cell lines in the BCoV/Resp infected group.

In MDBK cells, CEACAM1 mRNA expression was downregulated in both infected groups, with a more prominent downregulation observed in the BCoV/Ent infected group (Figure 15E). In BEC cells, CEACAM1 mRNA expression was significantly downregulated in the BCoV/Ent group but significantly upregulated in the BCoV/Resp group compared to the sham (Figure 15F).

Bovine APN mRNA expression was downregulated in the BCoV/Ent infected group in MDBK cells, while no notable change was observed in the BCoV/Resp infected group (Figure 15G). Conversely, BEC cells’ bovine APN mRNA expression was significantly upregulated in both BCoV-infected groups. Notably, a more obvious upregulation was observed in the BCoV/Resp infected group than in the sham group (Figure 15H).

The bovine DPP4 mRNA expression was significantly upregulated in BCoV/Resp infected groups, with no change observed in the BCoV/Ent infected group compared to the sham (Figure 15I). In BEC cells, bovine DPP4 mRNA expression was significantly upregulated in the BCoV/Ent group, while no significant change was observed in the BCoV/Resp infected group compared to the sham (Figure 15J).

In MDBK cells, bovine AXL mRNA expression was significantly upregulated in BCoV/Resp infected groups, while no change was observed in the BCoV/Ent infected group compared to the sham (Figure 15K). Similarly, bovine AXL mRNA expression in the BEC cells was significantly upregulated in BCoV/Resp infected groups. At the same time, no change was observed in the BCoV/Ent infected group compared to the sham (Figure 15L). These findings indicate that bovine AXL is specifically upregulated in response to BCoV/Resp infection in both cell lines, highlighting its role in the context of the BCoV/Resp isolates.

### 2.14. Differential Expression Pattern of Protease Enzymes upon BCoV/Ent and BCoV/Resp Infected Cells In-Vitro

**Figure 16:**
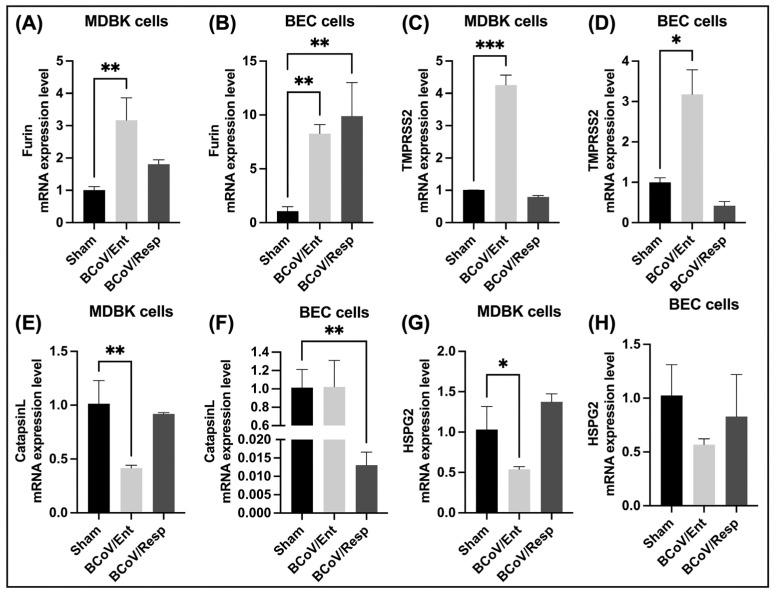
Assessment of Bovine (Furin, TMPRSS2, CTS-L, and HSPG2) mRNA expression profiles in BCoV infected MDBK and BEC cells using qRT-PCR. (**A**,**C**,**E**,**G**) Compared to the sham, Bovine Furin, TMPRSS2, CTS-L, and HSPG2 mRNA expression in MDBK cells infected with BCoV/Ent or BCoV/Resp isolates was analyzed by qRT-PCR. (**B**,**D**,**F**,**H**) Compared to the sham, Bovine Furin, TMPRSS2, CTS-L, and HSPG2 mRNA expression in BEC cells infected with BCoV/Ent or BCoV/Resp isolates analyzed by qRT-PCR. Both cell lines were infected with 1 MOI of either BCoV/Ent or BCoV/Resp isolates for 72 hpi and used for qRT-PCR analysis, (* *p* < 0.05, ** *p* < 0.01, *** *p* < 0.001).

In MDBK cells, bovine Furin mRNA expression was upregulated in both infected groups, with a significant increase observed in the BCoV/Ent infected group (Figure 16A). Similarly, bovine Furin mRNA expression in BEC cells was significantly upregulated in both infected groups (Figure 16B). This indicates that bovine Furin expression is elevated in response to BCoV infection, regardless of the isolate.

In MDBK cells, bovine TMPRSS2 mRNA expression was significantly upregulated in BCoV/Ent infected groups, while no change was observed in the BCoV/Resp infected group compared to the sham (Figure 16C). Similarly, in BEC cells, bovine TMPRSS2 mRNA expression was significantly upregulated in BCoV/Ent infected groups, while no change was observed in the BCoV/Resp infected group compared to the sham (Figure 16D). These findings indicate that bovine TMPRSS2 expression is elevated explicitly in response to BCoV/Ent infection.

The bovine CTS-L mRNA expression was significantly downregulated in BCoV/Ent infected groups, while no change was observed in the BCoV/Resp infected group compared to the sham (Figure 16E). In BEC cells, bovine CTS-L mRNA expression was significantly downregulated in BCoV/Resp infected groups, while no change was observed in the BCoV/Ent infected group compared to the sham (Figure 16F).

In both MDBK and BEC cells, bovine HSPG2 mRNA expression was downregulated in BCoV/Ent infected groups, while no change was observed in the BCoV/Resp infected group compared to the sham (Figure 16G,H).

### 2.15. Expression Pattern of Host Cell Surface Receptors and Enzymes in Bovine Lungs Tissues

**Figure 17:**
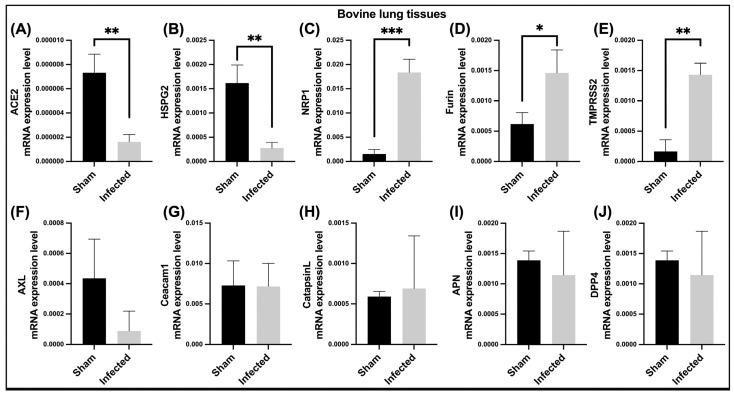
Assessment of Bovine receptors and enzymes mRNA expression in BCoV infected and control lungs in-vivo. (**A**–**J**) Bovine ACE2, HSPG2, NRP1, Furin, TMPRSS2, AXL, Ceacam1, CTS-L, APN, and DPP4 mRNA expression in bovine lungs collected from BCoV-infected and non-infected (sham) was analyzed by qRT-PCR. The mRNA expression level shows the 2-^CT values for both groups, (* *p* < 0.05, ** *p* < 0.01, *** *p* < 0.001).

To investigate the mRNA expression of bovine host cell surface receptors and enzymes associated with BCoV infection in vivo, qRT-PCR was performed on lung samples collected from non-infected (sham) and BCoV-infected calves. Results showed very low mRNA expression of ACE2 in both the sham and infected groups (Figure 17A). The mRNA expression of ACE2 and HSPG2 was significantly downregulated in bovine-infected lungs compared to the control (Figure 17A,B). The NRP1, Furin, and TMPRSS2 mRNA expression levels were significantly upregulated in bovine-infected lungs compared to the control (Figure 17C–E). In contrast, the mRNA expression of AXL, Ceacam1, CTSL, APN, and DPP4 did not show any significant differences between the BCoV-infected and control groups (Figure 17F–J).

## 3. Discussion

The tropism of coronaviruses is a complex process that involves factors including some attachment proteins, particularly spike glycoprotein. This process also involves some cellular factors, including the cellular receptors and other transcription and translation factors [29,30]. BCoV possesses multiple tissue tropism in cattle. The virus mainly affects the digestive and respiratory tract of the affected animals; this pattern is called pneumoenteritis [31]. The viral tropism primarily depends on the availability of specific viral receptors, some other transcription translation factors, and some host cell enzymes [32]. Members of the family coronaviridae utilize many host cell receptors to attach to their target cells [33].

Coronaviruses require activation by some host cell proteases to initiate the viral infection inside the host. Usually, the coronaviruses spike glycoproteins cleaved by some host cell proteases of different classes to initiate the viral infection [29,30]. Although BCoV was discovered long ago, little is still known about viral tropism, especially the roles of the host cell receptors and the host enzymes in fine-tuning the viral tissue tropism [31]. The main aims of the current study are to explore the possibilities of identifying some novel receptors of BCoV/S and to predict the interaction of some key viral proteins with some host proteases, particularly the Furin CTS-L and the TMPRSS2. The BCoV/S glycoprotein is the main viral protein involved in viral attachment to the host cells [34]. The S protein comprises two subunits (S1 and S2): the N-terminal domain (NTD) and BCoV_S1_CTD are in the S1 subunit, whereas the fusion peptide (FP) and heptad re-peat (HR.) domains 1 and 2 are located in the S2 subunit of the BCoV/S glycoprotein. The BCoV/S1 protein usually attaches to the cell membrane by interacting with viral receptors on the surface of the target cells, initiating the viral infection. Spike protein S2 mediates the fusion of the virion and cellular membranes by acting as a class I viral fusion protein. Also, it acts as a viral fusion peptide, which is unmasked following the S2 cleavage site occurring upon virus endocytosis [35,36]. The distal S1 subunit of the coronavirus spike protein is responsible for receptor binding. Either the S1-NTD or the S1-RBD at the C-terminal domain of the BCoV/S protein chain, or occasionally both, are involved in the binding to the host receptors [37,38].

Our docking (molecular interaction) result of the BCoV/S and the BCoV/HE proteins with the known BCoV receptors, Neu5,9Ac2, showed high binding affinity towards both the proteins of BCoV. However, based on binding energy, HE interaction with receptor Neu5,9Ac2 (−122.73Kcal/mol) is more stable and more significant compared to the interaction of the BCoV/S protein with the Neu5,9Ac2 (−79.55 kcal/mol). This suggests that HE may help in the interaction of BCoV with or without spike protein to bind to its specific cellular receptor Neu5,9Ac2. The BCoV-HE could interact with its cellular receptors and may cause changes to the cell surface, which could lead to infection of the host cells. BCoV/S and BCoV/HE proteins act synergistically and harmoniously to orchestrate the BCoV infection in the target cell [9]. The BCoV/S is mainly involved in the initial attachment of the virus to the host cells, while the BCoV/HE destroys the sialic acid in the cell’s surface, promoting the viral release from the host cells [9].

It has been proved that SARS-CoV-2 uses the ACE-2 as a valid receptor for the viral entry into the target host cells [39,40]. In our study, based on the interaction between bovine ACE2 and BCoV/S interaction with its CTD, the bovine cell surface ACE2 could act as a putative receptor for BCoV/S. Additionally, we demonstrated that bovine ACE2 is highly expressed in bovine kidney cells (MDBK) following BCoV infection, whereas ACE2 mRNA expression is downregulated in bovine endothelial cells (BEC) under the same condition (Figure 15A,B). This finding is based on the BCoV spike binding to the ACE2 receptor’s tendency to show lower interaction energy [41]. This claim is also supported by the nature of the interaction energy, which is consistent with the structure of the virus receptor interface [42]. Our previous studies showed the interaction between the BCoV and some host cell proteins, particularly ACE2. TMPRSS2 and NRP1 [43].

We also investigated the other bovine cell membrane protein Neuropilin1 (NRP1) as a receptor for the BCoV. Our in-silico protein-protein interaction result suggested a high interaction affinity of NRP1 with BCoV/S near the S1/S2 cleavage site. (Figure 6) and (Table 2). This raises the possibility that BCoV could use the NRP1 receptors for viral entry into the host cell. Furthermore, the in-vitro analysis revealed a significantly increased expression of NRP1 following BCoV infection (Figure 15C,D). However, the gene expression analysis showed that NRP1 mRNA expression was significantly elevated in BEC cells in both infected groups compared to the sham group (Figure 15C,D). The NRP1 expression was also upregulated in bovine lung tissues collected from the infected calves compared to those from non-infected calves (Figure 17C). Interestingly, NRP1 levels were elevated both in vitro and in vivo following BCoV infection. The higher affinity of NRP1 towards BCoV/S suggested that the NRP1 receptor protein was a therapeutic target. According to some previous reports, the higher expression of NRP1 will facilitate virus-host cell interactions, especially in cells that do not express other potential BCoV receptors [44,45]. Previous research indicates that NRP-1 exhibits stronger binding affinity to the CTD region of S1, and this interaction stabilizes the folded conformation of the S protein [46].

To observe the expression of other coronaviruses-related receptors, NGS was performed on BCoV-infected and control BEC cells. This investigation includes the expression of VEGF family member receptors, which are intact with NRP1 [47] and play a role in SARS-CoV-2 infection [48], as well as other receptors such as TMEM106 [49], SDC2 [50], and EPH-A/B receptors [51]. However, the NGS results only provide a comprehensive overview of the expression of these receptors and do not conform to their functional roles in BCoV infection. Therefore, further experimental investigation is required to elucidate the roles of these receptors in the context of BCoV infection.

The CEACAM-1 acts as a functional receptor for MHV and enhances the activation of the MHV-S glycoprotein by inducing some conformational changes, allowing the fusion of the virus with the target cells [52]. However, there is no data about the potential roles of CEACAM1 in the BCoV replication. Our data showing low binding affinity interaction between BCoV/S and CEACAM-1 suggested that CEACAM-1 interacted with low affinity to the NTD of BCoV/S protein. The in-vitro results further revealed downregulation of CEACAM1 mRNA expression in the BCoV/Ent isolate infected groups, whereas a significant upregulation was observed in the BCoV/Resp infected group (Figure 1 and Figure 15E,F). However, no significant differences were observed in the mRNA expression of CEACAM1 between control and infected lung samples (Figure 17G). The variations in the binding of coronaviruses to CEACAM1 can be directly attributed to the structural differences between their N-terminal domains, particularly those of BCoV and MHV. Unlike MHVs sugar-binding ancestors, contemporary MHVs utilize their NTDs for specific interaction with the host cell protein CEACAM1 [53].

The APN, a widely found cell surface molecule in animal cells, is involved in various cellular processes like survival, migration, blood pressure regulation, and even virus uptake [54]. Based on our protein-protein interaction (E_RDock), the interaction energy of the best pose of APN interaction with BCoV/S shows low-affinity interaction (−2.96 kcal/mol). This result confirms that the binding interaction of BCOV/S is not strong, and it shows a putative binding with the APN. The RBD of CTD from BCoV/S is involved in interaction with Bovine APN (Figure S5). Additionally, the in-vitro results showed that host APN was not expressed in MDBK cells following BCoV infection, but it was expressed in BEC cells (Figure 15G,H). However, no significant changes were observed in the mRNA expression of APN between control and infected lung samples (Figure 17I). This indicates that its activation may be limited to endothelial cells upon BCoV infection. PDCoV can utilize APN as a receptor to enter cells, highlighting APN’s potential role as a viral infection gateway from different host species [55]. Aminopeptidase N from porcine functions as a receptor for the enveloped RNA virus TGEV [12]. This underscores the wide variety of membrane-bound proteins viruses exploit to infiltrate cells. The APN is a common receptor for the members of the alpha coronaviruses, particularly the HCoV-229E and the TGEV in pigs [56,57,58].

The DPP4 is a cell surface protease that exhibits exopeptidase activity and is expressed on the surface of various cell types, including those found in human airways [59]. In our study, docking of the BCoV spike with the DPP4 shows low interaction affinity of DPP4 towards BCoV/S (E_R Dock score −6.23 kcal/mol). However, the binding of DPP4 with BCoV/S1-CTD region or S1B domain is correlated with MERS-CoV binding of its S1B domain or S1-CTD to the human DPP4 (Figure S5). Additionally, the mRNA expression of DPP4 was found to be specific to BCoV/Resp-infected MDBK cells, while in BEC cells, it was only expressed in the BCoV/Ent-infected group (Figure 15I,J). The DPP4 mRNA expression remained unchanged between control and infected lung samples (Figure 15J). This indicates an inconsistent pattern of bovine DPP4 expression in response to BCoV infection. The interaction studies between the receptor binding domain (RBD) of different spike variants of SARS-CoV-2 and DPP4 with the interactions observed in the experimentally determined structure of the MERS-CoV complex with DPP4 [60]. Members of the Betacoronaviruses, especially the SARS-CoV-2 and the MERS-CoV, utilize the ACE2 and DPP4 as receptors, respectively [61]. The pathogenicity of MERS-CoV is caused by the specific binding of its S1B domain or S1-CTD to the human DPP4 receptor [62].

The interaction study of NTD from BCoV/S with Bovine AXL (Figure S5), exhibited low binding affinity with NTD of BCoV/S with multiple non-covalent interactions, suggesting less possibility of its role in infection alone or with its synergistic effect with other receptors such as ACE2. The in-vitro experimental results support the in-silico prediction but indicate that AXL expression is specifically associated with infection by the BCoV/Resp isolate (Figure 1 and Figure 15K,L). The AXL mRNA expression remained unchanged between control and infected lung samples (Figure 17F). The in silico and in-vitro expression investigation results suggested AXL as a putative receptor but not the potent receptor for BCoV/S. The AXL receptor is found on the human cell membrane and contributes to the infectious process of SARS-CoV-2 [63]. AXL has been reported to bind to the NTD of the spike protein S1 subunit and could synergistically work with ACE2 to facilitate virus entry into cells [64]. Further, the NTD from BCoV/S is involved in interaction with HS from HSPG2 (Figure 17). HS shows binding affinity with NTD of BCoV/S due to multiple non-covalent interactions, suggesting the possibility of its role in infection with its synergistic effect with other receptors such as ACE2. The BCoV/S-HS interaction could interact with its other cellular receptors, such as ACE2, which may cause cell surface changes, leading to host cell infection. However, the in-vitro results showed downregulation of HSPG2 mRNA expression in both MDBK and BEC cells; bovine HSPG2 mRNA expression was downregulated in BCoV/Ent infected groups, while no change was observed in the BCoV/Resp infected group compared to the sham (Figure 16G,H).

For virus entry and infections, proteolytic cleavage is widely used to activate the fusion machinery of viral glycoproteins. In our study, the BCoV/S interaction with the Furin shows that Furin binds near the spike protein S1/S2 cleavage RRSRR site specific for Furin (Figure 11A). However, bovine Furin mRNA expression was upregulated in both MDBK and BEC cells infected, with a notable increase in the BCoV/Ent infected group (Figure 16A,B). Similarly, significant upregulation of bovine Furin mRNA was also observed in infected lungs (Figure 17D). This suggests that bovine Furin expression is heightened in response to BCoV infection, regardless of the isolate. Furin binding to RRSRR (Argi-nine-Arginine-Serine-Arginine-Arginine) site of BCoV spike protein, which is related to the conserved cleavage site of SARS-CoV-2 cleavable RRAR|S residues at receptor-binding (S1) and fusion (S2) domains of the spike protein [65]. Such a motif may allow Spikes to be cut into S1 and S2 by Furin and TMPRSS2-like proteases before maturity, which provides S1 with the flexibility to change the conformation to better fit the host receptor. The arginine residues at the SARS-CoV-2 spike protein catalytic site are popped out of the closed state of the S protein to form multiple non-covalent interactions with the Furin [66].

The protein-protein interaction occurs between the bovine TMPRSS2 amino acid residues and furin cleavage site RRSRR site of BCoV/S protein residues. The interaction of TMPRSS2 shows less binding affinity towards BCoV/S. The binding of TMPRSS2 near the S1/S2 junction RRSRR protease cleavage-specific region for furin protease might show cleavage at the S2′ site (CNKVSSRS) specific for TMPRSS2 protease of the spike protein (Figure 11B). The bovine TMPRSS2 mRNA was significantly upregulated in the BCoV/Ent infected group, with no changes in the BCoV/Resp group (Figure 16C). A similar pattern was observed in BEC cells (Figure 16D). Similarly, the mRNA expression of TMPRSS2 was also significantly up-regulated in bovine-infected lungs compared to the lungs from control calves (Figure 17E). These results indicate that bovine TMPRSS2 expression is specifically elevated in response to BCoV/Ent infection. TMPRSS2 has been shown to proteolytically activate the S glycoprotein of many coronaviruses, including SARS-CoV-2, SARS-CoV-1, MERS-CoV, 229E, as well as influenza virus [67,68,69]. TMPRSS2 triggers HKU1-mediated cell-cell fusion and viral entry and binds with high affinity to both HKU1A and HKU1B RBDs [70]. The protease furin binds firmly with the S protein RRSRR site, which is conserved in BCoV as RRSRR at the S1/S2 site, whereas the TMPRSS2 cleaves the S2′ protein in the lungs of SARS-CoV-2 infected person and promotes pathogenicity [71]. SARS-CoV-2 S protein S1/S2 is cleaved by furin protease, and subsequently, TMPRSS2 mediates the cleavage and activation of the S2 region on the S2 protein [72]. The first cleavage occurs at the S1/S2 boundary. This cleavage is typically facilitated by furin or other proprotein convertases. Furin is a cellular protease that recognizes a specific sequence in the S protein. Cleavage at this site results in the separation of the S1 subunit (which contains the receptor-binding domain) from the S2 subunit (which is involved in membrane fusion). The second cleavage occurs within the S2 subunit, specifically at the S2 site. This cleavage is often mediated by TMPRSS2, a serine protease exhibited on the surface of the host cell. Cleavage at this site is critical for activating the fusion machinery of the S protein, allowing the viral envelope to fuse with the host cell membrane and facilitate entry of the viral genome. TMPRSS2, which colocalizes with ACE2 at the cell membrane, has been identified as the dominant proteo-lytic driver of S protein activation and SARS-CoV-2 infection of the aerodigestive tract [73].

Protein CTS-L is a member of the lysosomal cysteine protease family. The expression level of CTS-L mRNA is higher than angiotensin-converting enzyme 2 (ACE2), Furin, and TMPRSS2 in human lung tissues [8]. In the context of SARS-CoV-2, the S protein contains two highly conserved CTSL cleavage sites (CS-1 and CS-2) among all known SARS-CoV-2 variants. CTSL cleavage promoted S to adopt receptor-binding domain (RBD) “up” activated conformations, facilitating receptor-binding and membrane fusion [9]. The highly conserved CS-2 site seems to be more essential for the life cycle of SARS-CoVs, while CS-1 is likely to play an auxiliary role in virus infection. However, with the help of multiple sequence alignment, it is observed that the BCoV/S has no conserved region for the CTS-L cleavage site as the SARS-CoV-2 sequence of the S chain. Based on our protein-protein interaction result, cathepsin showed binding to BCoV/S in NTD and downstream of the S1/S2 region of CTD of the S chain (Figure 14). However, the in-vitro expression study results showed a down-regulation of CST-L expression upon BCoV infection (Figure 14). The expression of CTS-L was significantly downregulated in BCoV/Ent infected groups, while no change was observed in the BCoV/Resp infected group compared to the sham (Figure 16E). Similarly, no significant changes were observed in the mRNA expression of CTS-L between control and infected lung samples (Figure 15H). This protein-protein interaction and expression analysis results of our study clearly suggest that CTS-L could play a putative receptor role in the infection of BCoV in the bovine hosts.

The results from our in-silico prediction investigation suggested that NRP1 showed greater binding affinity as a receptor towards BCoV/S. The results of the expression study analysis were also suggested and in agreement with the in-silico study to support NRP1 as a receptor of BCoV/S. In the case of protease enzymes, the furin and CTS-L bind with BCoV/S with greater binding affinity than TMPRSS2. However, our in silico structural interaction data provide a blueprint for understanding the BCoV specificity for the different bovine receptors. The expression analysis of Furin, TMPRSS2, and CTS-L reveals that the Furin expression and it’s in silico binding have high affinity towards BCoV/S, pointing to furin as a potent protease receptor for BCoV/S protein. Therefore, Furin and NRP1 have greater bonding affinity and elevated expression in response to BCoV infection, irrespective of the isolate, suggesting both proteins play a key play in disease and pathogenesis in bovines.

## 4. Materials and Methods

### 4.1. Bovine Cell Lines, Tissue Samples and Viruses

Bovine Pulmonary Artery Endothelial Cells (BEC) (Cat. No. CRL-1733) were obtained from ATCC. (Manassas, VA, USA) Dr. Udeni B. R. Balasuriya, Louisiana State University, kindly provided the Madine Darby Bovine Kidney (MDBK) cells. Both cell lines were tested for Bovine Viral Diarrhea Virus (BVDV) and found negative. BEC cells were cultured in F12 media (ATCC, Cat. No. 30-2004), supplemented with 10% Horse Serum (HS) (Gibco; New York, NY, USA, Ref. No. 26050-088) and 1% 10,000 µg/mL streptomycin and 10,000 units/mL penicillin antibiotics (Gibco; Ref. No. 15140-122). MDBK cells were cultured in Minimum Essential Medium Eagle (MEME) media (Sigma-Aldrich, St. Louis, MO, USA, Cat. No. M0200-500ML), supplemented with 10% HS and 1% streptomycin and penicillin antibiotics. Both cell lines were incubated at 37 °C at 5% CO_2_ for subsequent culture. Dr. Philip Gauger kindly provided bovine lung tissues from BCoV-infected and control calves from the Veterinary Diagnostic Laboratory, Iowa State University (ISU-VDL). All the samples from control and infected calves were tested for BCoV presence through qRT-PCR [74]. Bovine coronavirus (BCoV) enteric isolate ‘Mebus’ [1] was obtained from BEI resources (NIAID, NIH, Manassas, VA, USA, Cat. No. NR-445). The BCoV respiratory isolate was kindly provided by Dr. Aspen Workman (Animal Health Genomics Research Unit, USDA, ARS., US. Meat Animal Research) [75].

### 4.2. The Next Generation Sequencing (NGS)

BEC cells were independently infected with either BCoV Enteric (BCoV/Ent) or BCoV Respiratory (BCoV/Resp) isolates at a Multiplicity of Infection (MOI) of 1. The cells were monitored for up to 4 days for the development of Cytopathic Effect (CPE). The cells were collected from sham and BCoV-infected groups, and total RNA was extracted using a total RNA extraction kit (QIAGEN) following the manufacturer’s instructions. RNA samples were submitted to LC Sciences (LLC, 2575 West Belfort Street, Houston, TX, USA) for the NGS. RNA library preparation was briefly performed using Illumina’s TruSeq-small-RNA-sample preparation protocols (Illumina, San Diego, CA, USA). Quality control and quantification of the DNA libraries were conducted using an Agilent Technologies 2100 Bioanalyzer High Sensitivity DNA Chip. Single end 50bp sequencing was carried out on Illumina’s Hiseq 2500 sequencing system following the manufacturer’s instructions. Differential expression of mRNAs based on normalized deep-sequencing counts was analyzed using various statistical tests, including the Fisher exact test, Chi-squared 2 × 2 test, Chi-squared n × n test, the student’s *t*-test, or ANOVA, depending on the experimental design. The Heatmap was generated based on the bovine gene expression profile obtained from the NGS data in control and infected groups. The Kyoto Encyclopedia of Genes and Genome (KEGG) pathway enrichment analysis was constructed from NGS data to identify biological pathways enriched within a set of genes in control and infected groups. The heatmap and KEGG diagrams were constructed using an online bioinformatical tool (https://www.bioinformatics.com.cn/en (accessed on 1 February 2025)).

### 4.3. Extraction of the Total RNAs and the qRT-PCR Protocol

Following the manufacturer’s instructions, the total RNA was isolated from MDBK and BEC control and the infected cells using TRIzol LS Reagent (Invitrogen, Waltham, MA, USA; REF. No. 10296010). RNA concentration was assessed using a Nano-Drop OneC (Thermo Fisher Scientific, Waltham, MA, USA). cDNA was synthesized from RNAs using a high-capacity reverse transcription kit (Applied Biosystems, Waltham, MA, USA; Lot. No. 2902953) following the manufacturer’s instructions. The qRT-PCR was performed using Power-Up SYBR Green Master Mix (Applied Biosystems, Lot. No. 2843446) on a QuantStudio3 System (Applied Biosystems). The oligos used for qRT-PCR were designed using the online software Primer3 [76]. The relative gene expression was normalized to the bovine b-actin following the 2-^^Ct analysis [77]. The oligos used in this study are listed in (Table 3).

### 4.4. Prediction of BCoV Spike (BCoV/S) and Host Cell Receptor Proteins

Homology modeling is a method to build predicted homology structures of proteins based on 3D confirmations of most identical template proteins. MODELLER tool of Biovia Discovery Studio v22.1.021297 was used to predict comparative modeling with the help of the build homology model method, and it was employed for the 3D model structure confirmation prediction of our query protein sequences [78]. The query protein sequences, including BCoV-Spike protein (BCoV/S) and the bovine ACE2, Furin, TMPRSS2, CTS-L, NRP1, DPP4, APN, CEACAM-1 and AXL can be retrieved and downloaded in FASTA format. The FASTA file formats contain protein sequences, which can be retrieved and downloaded from NCBI (https://www.ncbi.nlm.nih.gov/) and Uniprot databases (https://www.uniprot.org/). The protein sequence of BCoV-spike (BCoV-S) glycoprotein (Mebus strain (ID: P15777)) was downloaded from the UniProt database. The query sequences of other bovine host proteins including the ACE2 (ID: Q58DD0), Furin (ID: Q28193), the TMPRSS2 (ID: A2VDV7), the NRP1 (ID: E1BMX5), DPP4 (ID: P81425), the APN (ID: P79098), CEACAM-1 (ID: Q6VAN8) and AXL (ID: F1N0D3) were retrieved from UniProt database. The homology modeling procedure requires the alignment of the query sequences with template protein sequences from the BLAST (https://blast.ncbi.nlm.nih.gov/Blast.cgi?PROGRAM=blastp&PAGE_TYPE=BlastSearch&LINK_LOC=blasthome (accessed on 1 February 2025)). The query protein sequence aligned with 100 most identical template sequences with a maximum identity of 99% and minimum 30% in BLAST with BLOSSUM62 algorithm protocol. Five identical template sequences (identity: maximum 99-minimum 65%) were chosen to be aligned by load structure and alignment method. Further, the 3D-build homology model protocol was run to align template structures and predict query sequence 3D confirmation based on template proteins. To predict five different homology models for each query sequence, Discrete Optimized Protein Energy (DOPE) score was used to assess the quality of the generated different 3D protein models. The DOPE Score and PDF total energy score are for evaluating protein model accuracy. The more negative values of the DOPE score and positive PDF total energy, the more stable and accurate the predicted protein model. The best model having the lowest DOPE score was selected for further in silico computational studies [79]. The correspondence homology model is then energy minimized (add hydrogen and CHARMm force field) using the energy minimization method. All the generated 3D structures of the all-atom models were verified through the Ramachandran plot and Verify 3D model tool of Biovia Discovery Studio v22.1.021297. Ramachandran plot suggested the stability of structure based on amino acid residues located in the most favored, highly allowed, and allowed region or site of the plot. If the verified score results from the Verify 3D method of the model protein is higher than the verified expected low Score value, then the model is of acceptable quality. The closer the verified score result is to the verified expected high score value, the better the quality of the predicted protein structure model. The prediction of structure is further visualized through Biovia Discovery Studio v22.1.021297.

### 4.5. Mapping the BCoV-Spike Glycoprotein Structural Domains

InterPro, v103.0, is an international initiative that was conceived to streamline the efforts of the signature database providers (http://www.ebi.ac.uk/interpro/ (accessed on 1 February 2025)) and contains signatures for protein families, domains, or functional sites. It has important tools for the computational functional classification of the newly determined sequences that lack biochemical characterization [80]. The characterization of functional site definitions in the annotation and functional classification of uncharacterized sequences was done through the InterPro-EMBL-EBI domain (https://www.ebi.ac.uk/interpro/search/sequence/ (accessed on 1 February 2025)). The sequence-based searches are done using InterProScan (https://www.ebi.ac.uk/interpro/about/interproscan/ (accessed on 1 February 2025)) and the Expasy- PROSITE server (https://prosite.expasy.org/), which combines the search methods from the member databases. The Web interface allows text-based and sequence-based searches using a sequence retrieval system (SRS) [81]. InterPro is accessible for interactive use via the EBI Web server (http://www.ebi.ac.uk/interpro (accessed on 1 February 2025)), which can also be reached via each of the member databases [82].

### 4.6. Molecular Docking with CDOCKER

The protein structure of the BCoV/S N-terminal domain (NTD) (residues 15–298), the BCoV-HE protein structure file was downloaded in the PDB file format from the Protein Data Bank (PDB) (https://www.rcsb.org/). In the molecular docking process of BCoV proteins with the ligand, the N-terminal domain (NTD) of BCoV spike protein (PDB ID:4h14) and HE protein (PDB ID:3CL5) was used. The ligand used in docking is sugar molecule N-acetyl-9-O-acetylneuraminic acid (Neu5,9Ac2), the most common type of sialic acid, generally acts as the terminal sugar in cell surface glycans and polysaccharides, was taken in 3D-SDF file format from PubChem database (https://pubchem.ncbi.nlm.nih.gov/). On the other hand, heparan sulphate (HS) glycan (PubChem CID: 53477715), a compound from the bovine membrane receptor heparan sulphate proteoglycan 2 (HSPG2), was taken in 3D-SDF file format from PubChem database. This docking analysis typically involves examining the ligand-protein interactions and the visualization of the docked complexes. To describe the binding site in protein, interaction binding affinity predicted by calculation of binding energy (ΔG), is likely an internal step within Biovia Discovery Studio v24.1.0.321712.

### 4.7. Analysis of the Protein-Protein Interactions of Some Bovine Host Cell Receptors and the BCoV/S Glycoprotein

The protein-protein interaction molecular docking was performed using the ZDOCK docking method, followed by refining the best poses through E_RDOCK through BIOVIA, Discovery studio v22.1.021297. The ZDOCK method uses a fast Fourier transform to perform the 3D search of the spatial degrees of freedom between two proteins. The molecular docking approaches, which take two (or more) structures as input and predict their complex structure, are increasingly being used for this purpose [83]. In each run of the ZDOCK, we used the default angular sampling (3600 ligand rotations) and a single 2.8 GHz 64-bit Opteron processor with 64 GB available system RAM. For protein-protein complex, the total desolvation score is simply the sum of the ACE scores of all receptor-ligand atom pairs within a distance cutoff of 6 Å. The protein-protein interactions interface was visualized by using the Biovia Discovery studio. The protocol ran for 200 different interaction poses of protein-protein interaction with 60 clusters. Therefore, the ZDOCK docking result poses were further refined for the best pose with minimum or lowest docking interaction energy/binding affinity with the refinement method, E_R Dock. This E_Rdock refined 10 poses with the lowest E_RDock energy from protein-protein docking of 200 poses of ZDOCK with multiple conformations and calculates the weighted energies with the scoring function of E_RDock [E_RDock = E_Sol + beta + E_elec2], where E_RDock denotes the scoring function of E_RDOCK, E_vdw1 and E_vdw2 denotes van der Waals non-bond interaction energy of the protein complex after the first and second CHARMm minimization, E_elec1 and E_elec2 is an electrostatic energy term of the protein complex after the first and second CHARMm minimization, and E_Sol represents desolvation energy of the protein complex calculated by ACE method [83]. The best pose was selected on the basis of its best (positive) score of ZDOCK and E_Rdock (negative) score. The best pose for blind docking from ZDOCK server output files can be selected based on your binding site and largest cluster choice. The resulting poses from the ZDOCK docking method must be further refined with the pose energy refined docked method (E_RDock) which is step after ZDOCK method to refine the best pose in the Biovia discovery studio. This pose was further used to analyze both proteins’ interaction site [84].

### 4.8. Statistical Analysis

All the presented data in this study were expressed as means (± S.D.) and were analyzed with GraphPad Prism v9 (https://www.graphpad.com/scientific-software/prism/www.graphpad.com/scientific-software/prism/ (accessed on 1 February 2025)). The one-way analysis of variance (ANOVA), along with Tukey’s or Dunnett’s post hoc test, was used to compare the results of multiple groups. Unpaired *t*-tests were used to compare statistical analysis between two groups. The (*p* values less than 0.05) were considered statistically significant. Statistical significance in the figures is shown as follows (* *p* < 0.05, ** *p* < 0.01, *** *p* < 0.001, **** *p* < 0.0001).

## 5. Conclusions

Our proposed in-silico modeling and docking show the potential interaction between the BCoV/S protein and some known coronavirus receptors (ACE2, DPP4, NPR1, APN, AXL and CEACAM-1) at various degrees. Therefore, based on the in-silico protein-protein interaction prediction result and expression analysis study from enteric and respiratory cell lines infection, NRP-1 might play the role of co-receptor for BCoV entry and pathogenicity of the virus into the bovine host cell. However, other than receptors, the enzyme proteases such as TMPRSS2, Furin, and CTS-L, our data confirms that the host cellular proteases (Furin and TMPRSS2) have conserved recognition sites within the BCoV/S glycoprotein. Furin and CTS-L showed higher binding affinity towards NTD of the S1 chain, and Furin showed higher binding affinity towards the S1/S2 junction of the S chain of the spike. The bovine furin mRNA expression was significantly upregulated in both infected groups. Combining in-silico predictions with in-vitro expression analysis provided a robust dual-layered methodology. This integration ensured the computational predictions were biologically relevant and validated under experimental conditions. The study highlighted NRP1 as a potential co-receptor for BCoV, paving the way for targeted further investigations of its mechanistic pathways that play a role in viral entry and pathogenesis in bovine hosts. Therefore, NRP1, TMPRSS2, and furin protease could be therapeutic targets for developing a drug against BCoV/Resp infection. Further, our findings offer essential insights for the further development of antiviral agents and expand the reservoir of anti-BCoV/S agents for targeting bovine coronavirus and other related coronaviruses.

## 6. Limitations of the Current Study and Gap in This Field of Research

In this study, we used a combination of molecular docking tools and gene expression profiles to identify potential host cell receptors and enzymes that could be involved in BCoV replication, particularly during viral entry. There are several limitations to the current study. First, their research in the field of BCoV receptors is minimal compared to other beta coronaviruses, especially the human coronaviruses such as (HCoV-HKU1, SARS-CoV-1, MERS-CoV-2, and SARS-CoV-2). Second, there is a lack of relevant research tools involved in the process of receptor validation, particularly the bovine antibodies, chemical inhibitors, and monoclonal antibodies. Third, the lack of the crystal structure and PDP files of some key BCoV proteins in the public domains makes the prediction and simulation challenging because we must develop a new model for each protein, which is a very lengthy procedure. Fourth, there is a lack of funding to support the research on some animal coronaviruses compared to other human coronaviruses. Fifth, the lack of a small animal model for BCoV makes this research very expensive if carried out using the actual host as an in vivo model.

## 7. Future Research Directions

We believe this study is going to pave the way for more research in this field of research in several aspects. First, more in vivo studies are required to validate the roles of the studied host cell receptors and enzymes in this study and other potential candidates further. Second, the development of some knock-out bovine cell lines will greatly improve the research in this direction and help in the validation of these potential receptors and enzymes in BCoV replication. Third, the development of some monoclonal antibodies or nanobodies against these BCoV potential receptors and enzymes will enhance the research in this field of study. Fourth, identification of the exact binding sites of the BCoV with their interactive sites in the host cell receptors may help in the design of some novel vaccines that protect cattle against the currently circulating BCoV strains in the field.

## 8. Clinical Implications of the Current Study

Identifying some novel BCoV host cell receptors and enzymes involved in the virus replication has many clinical implications. First, targeting the viral receptors using some chemical compounds will help in the design of some antiviral receptors that block the viral entry into the host cell; thus, the viral infection could be mitigated at the early stages of viral infection. Second, identification of the roles of the host cell enzymes, such as (Furin, TMPRRSS2, Cathepsins, etc) in the BCoV replication will help in the design of some compounds that inhibit the action of these enzymes and stop the viral replication. Third, Identifying the binding sites of the host cell receptors to the BCoV/S and BCoV/HE proteins will help design some monoclonal antibodies or nanobodies that block this interaction, thus inhibiting the downstream virus replication. Fourth, most of the generated data on the BCoV receptors and enzymes will have a significant impact on human health from the One Heath perspective due to the close relationship of BCoV to other human coronaviruses, particularly the HCoV-OC43 and other members of the genus Betacoronaviruses.

## Figures and Tables

**Table 1:** List of the homology modeling verification parameters.

Homology Model (Low— DOPE Score)	Verify Score	Verify Expected High Score	Verify Expected Low Score
**BCoV/S**	476.141	561.414	252.636
**ACE2**	308.35	358.11	161.153
**Furin**	227.99	216.48	97.4159
**TMPRSS2**	142.08	152.573	68.6579
**NRP1**	125.32	159.001	71.5506
**DPP4**	308.11	333.627	150.132
**APN**	385.36	415.465	186.959
**CEACAM-1**	32.41	58.3052	26.2373
**AXL**	135.46	137.42	61.84
**CTS-L**	166	155	34.77

**Table 2:** List the energy and bonding interactions between the BCoV/S glycoprotein, some potential bovine host cell enzymes, and some potential bovine cellular receptors.

S.No	Best Pose	ZDock Score	E_R Dock Score	Total Hydrogen Bonds	Total Pi Bonds	Salt Binding
1	BCoV/S-ACE2	22	−6.26	7	28	0
2	BCoV/S-NRP1	23	−15.22	29	9	0
3	BCoV/S-CEACAM-1	16.8	−6.29	12	9	1
4	BCoV/S-APN	17.6	−2.95	19	2	1
5	BCoV/S-DPP4	17.6	−2.95	19	2	1
6	BCoV/S-AXL	17.56	−13.68	21	9	0
7	BCoV/S-Furin	16.6	−13.14	30	1	0
8	BCoV/S-TMPRSS2	20	0.641	15	6	0
9	BCoV/S-CTS-L	19.84	−16.45	15	2	0

**Table 3:** List of the primers used to measure the gene expression profiles of some bovine host genes during BCoV infection in cell culture.

Bovine Genes	Forward Primer (5′ to 3′)	Reverse Primer (5′ to 3′)
**ACE2**	GCTGTCGGGGAAATCATGTC	TCTCTCGCTTCATCTCCCAC
**Furin**	CGAGAAGAACCACCCAGACT	CTACGCCACAGACACCATTG
**TMPRSS2**	CCTTCTTAGCAGCCCAGAGT	CATCTTCAAGGGAGGCCAGA
**NRP1**	CCAGAAGCCAGAGGAGTACG	GCCTTTTCCGATTTCACCCT
**CEACAM1**	TTCTTCTGCTTGCCCACAAC	TCCTTTGTAACGAGCAGGGT
**DPP4**	AGAGACGCAGACCATGAAGA	TCGGCTAGAGTGTAGGTTCTG
**AXL**	TCTCAGATGCGGGATGGTAC	AGCTCAGGTTGAAGGGAGTG
**APN**	AGAGTGGGACTTTGCTTGGA	TGGCAATGCTGGTAATGGTG
**HSPG2**	GTTGTCAGCGTGGTGTTCAT	GAGAGGTGACGTAGGAGGC
**CatapsinL**	CTTCGATTCCTCCATCCGTG	TCTATGAAGCCACCGTGACA
**β** **-actin**	CAAGTACCCCATTGAGCACG	GTCATCTTCTCACGGTTGGC

## Data Availability

The authors will make the raw data supporting this article’s conclusions available upon request.

## Supplementary Materials

The following supporting information can be downloaded at: https://www.mdpi.com/article/10.3390/ijms26031328/s1.
