# Supplementary material for: Leveraging Artificial Intelligence and Gene Expression Analysis to Identify Some Potential Bovine Coronavirus (BCoV) Receptors and Host Cell Enzymes Potentially Involved in the Viral Replication and Tissue Tropism
Source: Int J Mol Sci. 2025 Feb 4;26(3):1328. doi: 10.3390/ijms26031328 (PMC11818245; doi:10.3390/ijms26031328)
Supplement: Supplementary file 1 [file ijms-26-01328-s001.zip › ijms-3414254-supplementary.pdf]

Supplementary Data

The Homology Modelling for the BCoV-Spike glycoprotein

Table S1. Top Homology Models by Sequence ID Based on DOPE Score and PDF Total Energy

Sequence UniProt ID	Bovine Protein Receptors	Homology Model	PDF Total Energy	DOPE Score
**P15777**	BCoV-Spike	Model 2	51989.78	-135770.54
		Model 1	53052.81	-133727.64
**Q58DD0**	ACE2	Model 1	34827.88	-94345.04
		Model 2	34765.74	-94330.17
**Q28193**	Furin	Model 2	16666.25	-53696.58
		Model 1	16719.49	-53651.78
**A2VDV7**	TMPRSS2	Model 1	11,639.4	-34,427
		Model 2	11,659.4	-34,507
**A0A3S5ZPJ8**	Cathepsin-L	Model 1	3415.4	-33161
		Model 2	3333.23	-32112
**E1BMX5**	NRP1	Model 2	15313.95	-25423.16
		Model 1	15491.51	-25920.80
**P81425**	DPP4	Model 1	33378.53	-90256.60
		Model 2	33326.76	-89901.53
**P79098**	APN	Model 1	35879.33	-118318.42
		Model 2	35532.13	-118316.10
**Q6VAN8**	CEACAM-1	Model 5	5361.93	-10424.35
		Model 2	5319.78	-9986.85
**F1N0D3**	AXL	Model 1	20221.22	-35499
		Model 2	19221.22	35122

Prediction of BCoV spike (BCoV/S) and host cell receptor proteins.

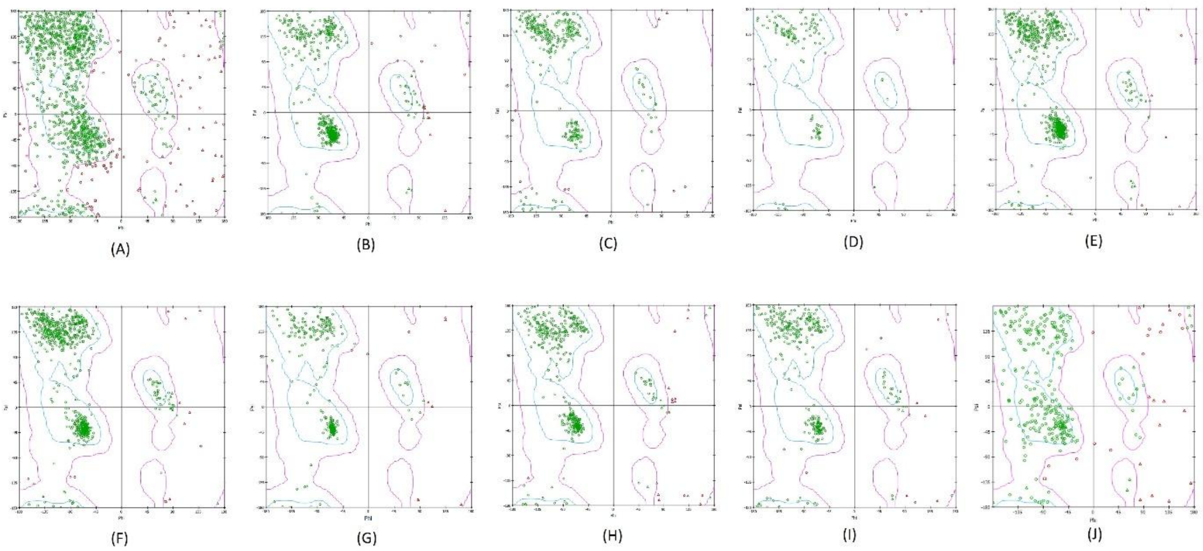

Figure S1. The validity and stability of the homology modeled protein structure using the Ramachandran plot (A) BCoV-Spike (B) Bovine ACE2 (C) Bovine NRP1 (D) Bovine CEACAM-1 (E) Bovine APN (F) Bovine DPP4 (G) Bovine AXL (H) Bovine Furin (I) Bovine TMPRSS2 (J) Cathepsin-L.

```
sp_P15777_SPIKE_CVBM   767    STKRRSR|RA  0.289    .
sp_P15777_SPIKE_CVBM   768    TKRRSRR|AI  0.774 *ProP*
sp_P15777_SPIKE_CVBM   775    AITTGYR|FT  0.085    .
```

A

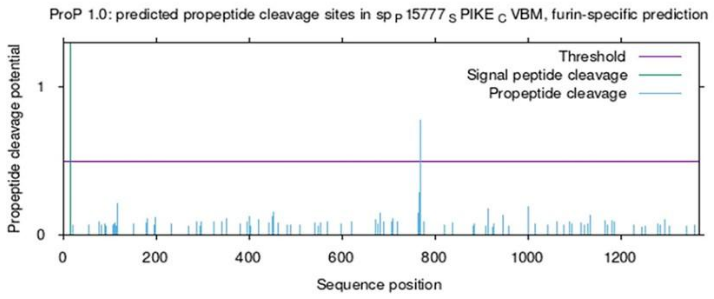

B

Figure S2. The ProP 1.0 Server software prediction of the Propeptide cleavage site (RRSRR|A) amino acids residues present in the full-length sequences of the BCoV/S protein

Table S2: The interacting residues between the BCoV-Spike protein and bovine ACE2, NRP1, CEACAM, APN, and DPP4 protein predicted by ZDOCK method of Biovia, Discovery studio.

BCoV/S-ACE2	BCoV/S-NRP1	BCoV/S-CEACAM1	BCoV/S-APN	BCoV/S-DPP4
:ARG419 - :GLU37	:THR422 - :LEU12	:ARG143 - A:LEU233	:ASN397 - :CYS826	:TRP184 - :ASP451
:GLN18 - :ASP608	:ASN432 - :ASP250	:ARG143 - A:PHE232	:TYR451 - :ASP849	:HIS185 - :ASP451
:HIS34 - :CYS530	:ARG1077 - :SER47	:ASN170 - A:VAL229	:ARG514 - :GLU843	:TRP186 - :ASP500
:THR82 - :ASP662	:ARG1077 - :TYR48	:THR171 - A:PHE232	:ARG514 - :SER839	:LYS196 - :LEU448
:SER84 - :ASN664	:LEU1081 - :ALA21	:ASN178 - A:THR145	:LYS579 - :ILE512	:TRP401 - :ASN146
:GLN324 - :ILE512	:GLN1084 - :ALA19	:LYS196 - :TRP154	:LYS579 - :GLY511	:LYS422 - :THR144
:ASN329 - :THR519	:ARG1089 - :GLN6:	:ARG197 - :ARG143	:ASN583 - :PRO516	:LYS422 - :GLU182
:THR333 - :ALA517	:ARG80 - :GLY278	:ASP225 -: TYR173	:CYS524 - :ASN495	:ARG452 - :HIS185
:ASP354 - :ASN526	:ARG80 - :ASP277	:LYS231 - :ASN170	:CYS524 - :ASN576	:ARG452 - :THR188
:ARG419 - :ALA385	:HIS130 - :ASN235	:HIS156 - :GLN141	:SER827 - :ASN397	:ARG452 - :GLY189
:THR425 - :GLU30	:TRP214 - :ASP370	:LYS150 - :TRP175	:LEU841 - :GLU398	:LYS512 - :LYS179
:THR514 - :ASP328	:ARG307 - :TYR237	:LYS150 - :TRP176	:SER846 - :GLN517	:LYS522 - :ASN175
:CYS515 - :GLU304	:SER1076 - :TYR44:	:ARG197 - :TRP215	:PHE847 - :GLN517	:LYS522 - :PRO174
:ASN520 - :ASP328	:LYS3 - :GLU1082	:GLU156: - :HIS156	:PRO515 -: GLN840	:LYS522 - :ASN175
:THR523 - :ASN329	:GLY20 - :GLN1084	:TRP215 - :TYR195	:CYS515 - :ASN583	:PHE533 - :THR23
:CYS524 - :ASN329	:HIS130 - :ASN235	:TRP154 - :LYS196	:GLY518 - :ALA555	:LEU183 - :ASP451
:ASP662 - :THR20	:GLY216 - :ILE367	:TRP176 - :LEU147	:SER516 - :TYR844	:GLY189 - :ASN495
:SER846 - :GLY574	:ARG197 - :TYR175	:ARG187 - :ASP148		:SER510 - :CYS21
:GLU30 - :THR423	:ARG1077 - :HIS49			:VAL24 - :PHE533

:HIS478 -<br>:GLN324<br>:THR519 -<br>:ASP328<br>:HIS607 -<br>:GLU30<br>:HIS607 -<br>:HIS34:<br>:SER611 -<br>:GLN24<br>:SER846 -<br>:GLU570<br>:LYS31 -<br>:HIS607<br>:GLU570 -<br>:PHE847<br>:SER19 -<br>:TYR661<br>:THR20 -<br>:TYR661<br>:ASN526 -<br>:TYR41<br>:ALA385 -<br>:LEU410<br>:ALA528 -<br>:LYS352<br>:HIS34 -<br>:CYS530	:LYS53 -<br>:PHE232<br>:GLN368 -<br>:PHE217<br>:VAL269 -<br>:TYR136<br>:HIS130 -<br>:TYR195<br>:TYR237 -<br>:VAL264<br>:HIS49 -<br>:ARG1077			:GLY475 -<br>:TRP186<br>:HIS143 -<br>:PRO450<br>:TRP184 -<br>:PRO450<br>:HIS185 -<br>:ARG452<br>:TRP186 -<br>:LEU476<br>:TRP524 -<br>:PRO174<br>:LYS179 -<br>:ASP514

Table S3: The interacting residues between the BCoV-Spike protein and bovine furin, CTS-L and TMPRSS2 protease protein predicted by ZDOCK method of Biovia, Discovery studio.

BCoV/S-Furin	BCoV/S-TMPRSS2	CTS-L-BCoV/S

:LYS763 - :ASP174<br>:LYS763 - :ASP177<br>:LYS763 - :ASP228<br>:ARG767 -<br>:GLU230<br>:TYR760 -<br>:MET189<br>:TYR760 -<br>:ASP191<br>:LYS763 - :ASP177<br>:GLY229 - :LYS763<br>:SER761 -<br>:ASN192 :LYS763 -<br>:ASP228<br>:SER766 -<br>:GLU230<br>:ARG764 -<br>:LEU227	:ASN739 -<br>:GLY462 :ASN739<br>- :CYS463<br>:GLN746 -<br>:PRO299<br>:ARG767 -<br>:ASP415<br>:ARG775 -<br>:LYS338<br>:GLN274 -<br>:GLU780<br>:GLU297 -<br>:SER743<br>:LYS304 - :SER786<br>:ARG325 -<br>:ASP789<br>:ARG325 -<br>:GLU792<br>:LYS340 -<br>:ARG764<br>:SER461 -<br>:THR771<br>:ARG468 - :ILE724<br>:ARG468 - :ILE724<br>:SER742 -<br>:VAL278 :LYS390 -<br>:TYR730<br>:LYS465 -<br>:TYR726,<br>:TRP459 - :ILE770	:LYS267-<br>:ARG768<br>:LYS267-<br>:ARG768<br>:LYS271-:PRO723<br>:LYS271-:GLN722<br>:LYS271-:PRO723<br>:THR275-:THR38<br>:ASP326-<br>:PHE713<br>:GLY190-:ALA33<br>:PHE186-:SER35<br>:TYR159-:ILE36<br>:ASP326-<br>:GLN722<br>:GLU142-<br>:ARG768<br>:GLU142-<br>:ARG768<br>:THR143-<br>:ARG768<br>:PRO149-:GLY72

(A)

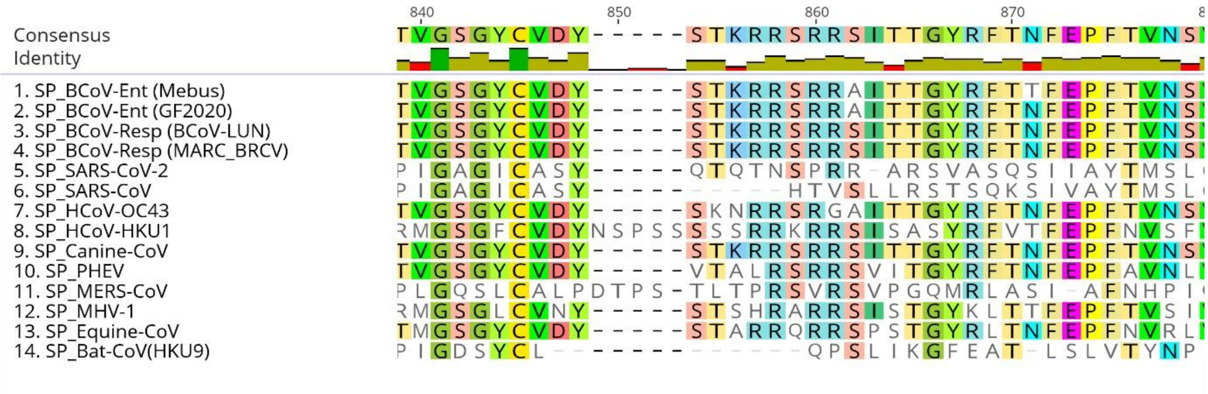

(B)

Consensus	GSGYCVDY-----STKRRSRRSITTGYRFTNFEPFTVNSVNDSLEPVG-GLYEIQIPSEF
SP_BCoV-Ent (Mebus)	GSGYCVDY-----STKRRSRRAITTGYRFTTFEPFTVNSVNDSLEPVG-GLYEIQIPSEF
SP_BCoV-Ent (GF2020)	GSGYCVDY-----STKRRSRRAITTGYRFTNFEPFTVNSVNDSLEPVG-GLYEIQIPSEF
SP_BCoV-Resp (BCoV-LUN)	GSGYCVDY-----STKRRSRRSITTGYRFTNFEPFTVNSVNDSLEPVG-GLYEIQIPSEF
SP_BCoV-Resp (MARC_BRCV)	GSGYCVDY-----STKRRSRRSITTGYRFTNFEPFTVNSVNDSLEPVG-GLYEIQIPSEF
SP_SARS-CoV-2	GAGICASY-----QTQTNSPRR-ARSVASQSIIAYTMSLGAEN--SVAYSNNSIAIPTNF
SP_SARS-CoV	GAGICASY----------HTVSLLRSTSQKSIVAYTMSLGADS--SIAYSNNTIAIPTNF
SP_HCoV-OC43	GSGYCVDY-----SKNRRSRGAITTGYRFTNFEPFTVNSVNDSLEPVG-GLYEIQIPSEF
SP_HCoV -HKU1	GSGFCVDYNSPSSSSSRRKRRSISASYRFVTFEPFNVSFVNDSIESVG-GLYEIKIPTNF
SP_Canine-CoV	GSGYCVDY-----STKRRSRRSITTGYRFTNFEPFTVNSVNDSLQPVG-GLYEIQIPSEF
SP_PHEV	GSGYCVDY-----VTALRSRRSVITGYRFTNFEPFAVNLVNDSIEPVG-GLYEIQIPSEF
SP_MERS-CoV	GQSLCALPDTPS-TLTPRSVRSVPGQMRLASI-AFNHPIQVDQLNS---SYFKLSIPTNF
SP_MHV-1	GSGLCVNY-----STSHRARRSISTGYKLTTFEPFTVSIVNDSVQSVG-GLYEMQIPINF
SP_Equine-CoV	GSGYCVDY-----STARRQRRSPSTGYRLTNFEPFNVRLVNDSVEPVG-GLYEIQIPSEF
SP_Bat-CoV (HKU9)	GDSYCL-------------QPSLIKGFEAT-LSLVTYNPLADSLTPIT-PVYQVSVPTNF

Figures S3: The sequence alignment of the (A) Bovine coronavirus (BCoV) enteric and respiratory strain spike protein sequence with spike protein sequences of other betacoronaviruses. (B) The red highlighted residues are Furin and TMPRSS2 specific cleavage site present at S1/S2 junction of spike protein. This polybasic residue (RRXRR) site presents in all the spike protein sequences (SP) of betacoronaviruses except the SARS-COV and Batcoronavirus (HKU9) strain.

Multiple Sequence alignment-Cathepsin-L (CTS-L)

(A)

```
Consensus                   TKFLFNVYLGTVLSHYYVM------PLTCNSALT------LEYWVT 271
SP_BCoV-Ent (Mebus)         TKFLFNVYLGTVLSHYYVL------PLTCSSAMT------LEYWVT 263
SP_BCoV-Ent (GF2020)        TKFLFNVYLGTVLSHYYVM------PLTCNSALT------LEYWVT 263
SP_BCoV-Resp (BCoV-LUN)     TKFLFNVYLGTVLSHYYVM------PLTCNSALT------LEYWVT 263
SP_BCoV-Resp (MARC_BRCV)    TKFLFNVYLGTVLSHYYVL------PLTCNSALT------LEYWVT 263
SP_SARS-CoV-2               LEPLVDLPIGINITRFQTLLALHRSYLTPGDSSSGWTAGAAAYYVG 268
SP_SARS-CoV                 LKPIFKLPLGINITNFRAI------LTAFSPAQDIWGTSAAAYFVG 255
SP_HCoV-OC43                TKFLFNVYLGMALSHYYVM------PLTCNSKLT------LEYWVT 267
SP_HCoV-HKU1                TTFLFSLYLGTLLSHYYVL------PLTCNAISSNTDNETLQYWVT 259
SP_Canine-CoV               TKFLFHVYLGTVLSHYYVM------PLTCNSVMT------LEYWVT 263
SP_PHEV                     TKFLFKLYLGTVLSHYYVM------PLTCDSALS------LEYWVT 263
SP_MERS-CoV                 DLYGGNMFQFATLPVYDTIKYYSIIPHSIRSIQSD-RKAWAAFYVY 316
SP_MHV-1                    TTFLFSIYIGDVLTQFFVL------PFNCEPDKA--GVISPQYWVT 262
SP_Equine-CoV               TTFLFNIYLGTSLSHYYVM------PLSCSGKLD------LQYWVT 263
SP_Bat-CoV(HKU9)            YLRIPAGFKPDVVQFFQAI-T----RPNGESAEVACASSTVSWYVS 288
```

(B)

```
Consensus                   RNJKCNYVF----------NNTLSRQLQPINYFDSYLGCVVNADNS 732
SP_BCoV-Ent (Mebus)         RNIKCNYVF----------NNTLSRQLQPINYFDSYLGCVVNADNS 740
SP_BCoV-Ent (GF2020)        RNIKCNYVF----------NNTLSRQLQPINYFDSYLGCVVNADNS 740
SP_BCoV-Resp (BCoV-LUN)     RNIKCNYVF----------NNTLSRQLQPINYFDSYLGCVVNADNS 740
SP_BCoV-Resp (MARC_BRCV)    RNIKCNYVF----------NNTLSRQLQPINYFDSYLGCVVNADNS 740
SP_SARS-CoV-2               QDVNCTEVPVAIH--ADQLTPTWRVYSTGSNVFQTRAGCLIGAEHV 656
SP_SARS-CoV                 QDVNCTDVSTAIH--ADQLTPAWRIYSTGNNVFQTQAGCLIGAEHV 642
SP_HCoV-OC43                RNIKCNYVF----------NNSLTRQLQPINYFDSYLGCVVNAYNS 730
SP_HCoV-HKU1                RNLKCSYVL-----------NNISLTTQP--YFDSYLGCVFNADNL 727
SP_Canine-CoV               RNIKCNYVF----------NNTLSRQLQPINYFDSYLGCVVNADNS 740
SP_PHEV                     RNLKCSHVF----------NNTVLGQIQLVNYFDSYLGCVVNAYNN 726
SP_MERS-CoV                 GSLACEHISSTMSQYSRSTRSMLKRRDSTYGPLQTPVGCVLGLVNS 720
SP_MHV-1                    RNLKCDYVF----------NNNISREETPLNYFDSYLGCVVNADNS 730
SP_Equine-CoV               RNLKCNYVF----------NNTIAREGNPINYFDSYLGCIVNADNS 740
SP_Bat-CoV(HKU9)            NNLPCSNRHRAV--V-EPVSAYWRRSVADNNTFDTTAGCIFNAYNL 648
```

Figure S4: The multiple sequence alignment of the Bovine coronavirus (BCoV) enteric and respiratory strain (highlighted green) spike protein sequence with spike protein sequences of SARS-COV-2 (highlighted yellow) and other betacoronaviruses spike protein sequences. (A) The red highlighted residues are conserved residues are Cathepsin-L specific CS-1 site. It is specific cleavage site present at NTD of S1 chain of BCoV/S. This Cathepsin-L specific CS-1 site is not present in all the spike protein sequences (SP) of betacoronaviruses except the SARS-COV2. (B) The red highlighted residues are conserved residues are Cathepsin-L specific CS-2 site. It is specific cleavage site present at NTD of S1 chain of BCoV/S. This Cathepsin-L specific CS-1 site is not present in all the spike protein sequences (SP) of betacoronaviruses except the SARS-COV-2.

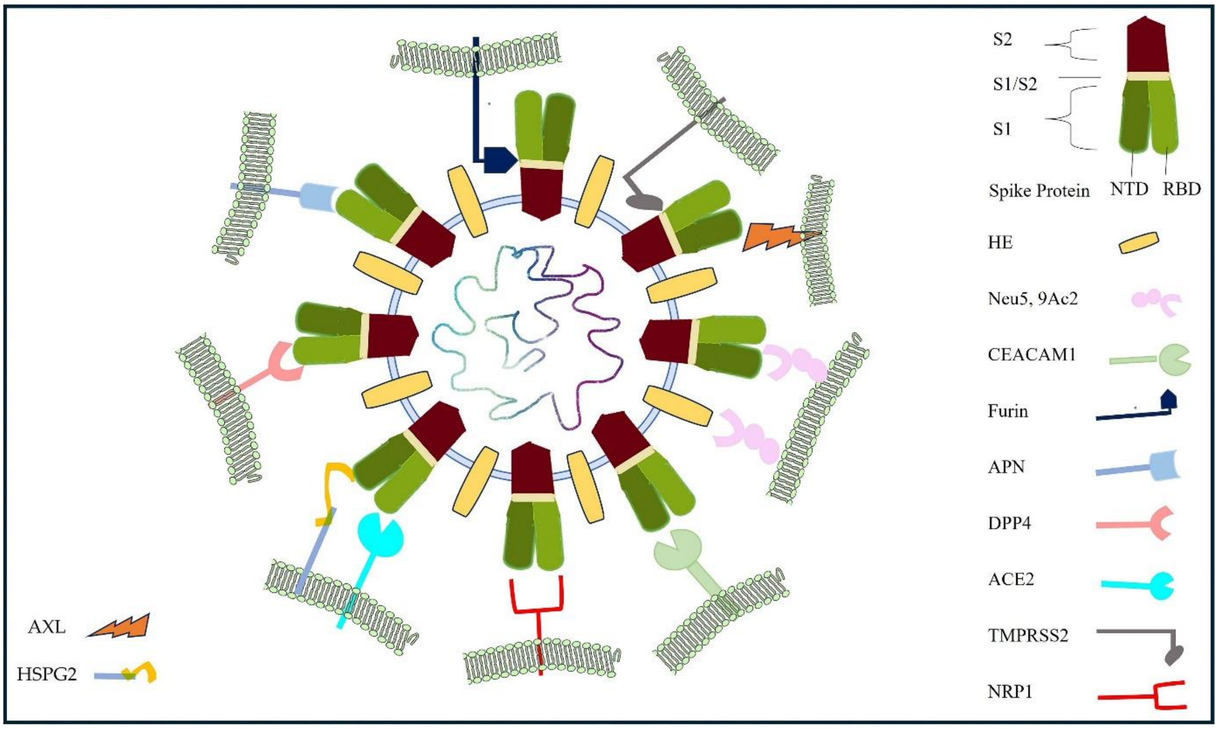

Figure S5. A schematic representation showing the interaction between the BCoV/S and BCoV/HE proteins with the potential cellular receptors, including host cell proteases (Furin and TMPRSS2). With most of the receptors, BCoV/S interacted with NTD and CTD. The NTD is an N-terminal domain present in the BCoV/S1 protein. The CTD is the C-terminal domain of the BCoV/S1 chain.
